# ­Characterization of pyruvate kinase from the anoxia tolerant turtle, *Trachemys scripta elegans*: a potential role for enzyme methylation during metabolic rate depression

**DOI:** 10.7717/peerj.4918

**Published:** 2018-06-08

**Authors:** Amanda M.S. Mattice, Isabelle A. MacLean, Christine L. Childers, Kenneth B. Storey

**Affiliations:** 1 Institute of Biochemistry, Department of Biology, Carleton University, Ottawa, Canada; 2 Institute of Biochemistry, Department of Biology and Chemistry, Carleton University, Ottawa, Canada

**Keywords:** Enzyme regulation, Anoxia, Glycolysis, Posttranslational modifications, Pyruvate kinase, *Trachemys scripta elegans*

## Abstract

**Background:**

Pyruvate kinase (PK) is responsible for the final reaction in glycolysis. As PK is a glycolytic control point, the analysis of PK posttranslational modifications (PTM) and kinetic changes reveals a key piece of the reorganization of energy metabolism in an anoxia tolerant vertebrate.

**Methods:**

To explore PK regulation, the enzyme was isolated from red skeletal muscle and liver of aerobic and 20-hr anoxia-exposed red eared-slider turtles (*Trachemys scripta elegans*). Kinetic analysis and immunoblotting were used to assess enzyme function and the corresponding covalent modifications to the enzymes structure during anoxia.

**Results:**

Both muscle and liver isoforms showed decreased affinity for phosphoenolpyruvate substrate during anoxia, and muscle PK also had a lower affinity for ADP. *I*_50_ values for the inhibitors ATP and lactate were lower for PK from both tissues after anoxic exposure while *I*_50_ L-alanine was only reduced in the liver. Both isozymes showed significant increases in threonine phosphorylation (by 42% in muscle and 60% in liver) and lysine methylation (by 43% in muscle and 70% in liver) during anoxia which have been linked to suppression of PK activity in other organisms. Liver PK also showed a 26% decrease in tyrosine phosphorylation under anoxia.

**Discussion:**

Anoxia responsive changes in turtle muscle and liver PK coordinate with an overall reduced activity state. This reduced affinity for the forward glycolytic reaction is likely a key component of the overall metabolic rate depression that supports long term survival in anoxia tolerant turtles. The coinciding methyl- and phospho- PTM alterations present the mechanism for tissue specific enzyme modification during anoxia.

## Introduction

The present study analyzes enriched pyruvate kinase (PK; 2.7.1.40) from the red-eared slider turtle, *Trachemys scripta elegans*, red muscle and liver tissues. Turtles of the *Chrysemys* and *Trachemys* genera are well-known to survive extended periods without oxygen by quickly and reversibly lowering their metabolic rate to avoid consuming more ATP than can be produced (Storey, 2007; Biggar, Groom & Storey, 2011). Metabolic rate depression during anoxia is crucial for survival otherwise anaerobic fuel in the form of glycogen would be quickly consumed (Biggar, Groom & Storey, 2011). As PK is a glycolytic control point that turtles rely on during the anaerobic catabolism of glycogen stores, analysis of PK kinetic changes helps to elucidate how reorganization of energy metabolism is coordinated throughout pathways and tissues to enable survival during oxygen restriction. Methylation is a budding area of interest in non-histone protein regulation and has been demonstrated to impact regulatory phosphorylation mechanisms by blocking phosphorylation sites. For example, FOXO1 methylation blocks phosphorylation by AKT resulting in the increased stability and transcriptional activity of FOXO1 (Yamagata et al., 2008). Methylation therefore may be an important agent in effecting protein function by regulating other post-translational modifications (PTMs) such as phosphorylation. This study evaluated the effects of 20-hr whole-animal anoxia exposures on PK properties and assessed the potential involvement of methylation and phosphorylation in contributing to stress-responsive PK control. Evidence of phosphorylation and methylation modifications each could provide novel mechanisms for tissue specific kinetic alterations to enzyme function.

It is well known that reversible PTMs can be used to control cellular metabolism in response to environmental conditions by influencing enzyme form and function through alteration of protein stability and substrate binding capacities (Humphrey, James & Mann, 2015). Recent work in our lab has provided evidence that this regulation can be clearly tissue specific within an animal to provide an even more nuanced response to stress, such as during anoxia tolerance in turtles. Previously, Xiong & Storey (2012) demonstrated that after 20-hr anoxia exposure lactate dehydrogenase (LDH), the terminal enzyme of anaerobic glycolysis, enriched from *T. s. elegans* liver is significantly more phosphorylated and acetylated, and is less active than normoxic controls. Dawson, Bell & Storey (2013) have also demonstrated that the muscle isoform is significantly dephosphorylated and de-acetylated, and likewise more active. Thus, both the turtle tissues use the removal of phosphorylation and acetylation as on/off-switch mechanisms in *T. s.elegans* in order to regulate the function of LDH. The liver tissue poises LDH to reduce anaerobic glycolysis, likely saving stored glycogen, while muscle poises it to promote anaerobic glycolysis, which combats increasing product inhibition.

PK catalyzes the final branch point of the glycolytic pathway as its product pyruvate is either shuttled to the mitochondria for oxidative phosphorylation or metabolised by LDH to form lactate. Unlike LDH, the PK reaction is not reversible. PK transfers the phosphate group from phosphoenolpyruvate (PEP) to ADP to produce ATP and pyruvate (Mattevi, Bolognesi & Valentini, 1996). This enzyme is therefore at a critical branch point of metabolism and plays a key role in regulating the mode of consumption of anaerobic fuels. Furthermore, multiple allosteric regulators can modulate PK activity, such as fructose −1,6-bisphosphate (FBP), phenylalanine and alanine (Tripodi et al., 2015). FBP is the central glycolytic metabolite produced by phosphofructokinase and is an allosteric activator of PK activity by increasing PEP binding affinity and stabilizing the enzyme tetramer in the active state (Mattevi, Bolognesi & Valentini, 1996; Tripodi et al., 2015). Therefore, the response to PTM regulation in turtle PK isoforms could be even more nuanced when metabolites are present.

## Methods and Materials

### Animals

Local suppliers provided adult female red-eared sliders (*T. s. elegans*) weighing 700–1,500 g. The turtles were held at 5 ± 1 °C in large plastic tubs filled with dechlorinated tap water for a week with two turtles per tub. Control turtles were sampled after this period. The remaining turtles were transferred into large buckets with water (at 5 ± 1 °C) that had been previously bubbling with nitrogen gas for one hour. Two or three turtles were added in 30-minute intervals and bubbling with N_2_ was continued until 1 hr past addition of the last turtle. Bubbling was reinitiated during the subsequent sampling of the animals after 20-hr of anoxic submergence. To ensure turtles remained submerged, a wire mesh was fitted into the tank approximately 5 cm below the water surface. All animals were killed by decapitation and tissues were immediately excised, frozen in liquid nitrogen and stored at −80 °C until use. All animals were cared for in accordance with the guidelines of the Canadian Council on Animal Care and all experimental procedures had the prior approval of the Carleton University Animal Care Committee (106937).

### Preparation of tissue extracts

Samples of frozen red muscle or liver were separately homogenized in a 1:10 w:v in ice cold Buffer A at pH 6.0 (10 mM MES, 2 mM EDTA, 2 mM EGTA, 15 mM β-glycerophosphate, 15 mM β-mercaptoethanol, 10% v/v glycerol) in the presence of a few crystals of phenylmethylsulfonyl fluoride (PMSF). Tissue homogenates were then centrifuged at 13,500× g at 4 °C for 30 min. The supernatant was collected and held at 4 °C until use.

### Pyruvate kinase purification

#### Red muscle

PK was enriched from red muscle in a single step procedure. Red muscle extract was applied to a CM^−^ Sephadex G50 column (1 × 4 cm) equilibrated with buffer A. Unbound protein was removed with a 30 mL wash with buffer A while collecting 2.5 mL fractions. The target enzyme was eluted with 30 mL of a 0-15 mM PEP gradient while collecting 1.6 mL fractions. Activity was evaluated in each fraction with 10 µL aliquots. PEP was then removed from the enriched sample using 5 ml of G-25 Sephadex (Cat# G25150-10G, Sigma-Aldrich). For every 500 µL of enzyme sample, 5 mL of beads equilibrated with 5 mL of buffer A were used and centrifuged at 2,500× g for 1 min.

#### Liver

PK was partially enriched from liver tissue using a two-column procedure. A sample of liver extract supernatant was applied to a CM^−^ Sephadex G50 column (1 × 4 cm) previously equilibrated with 30 mL of buffer A. Application of the enzyme sample was followed by a 30-mL wash with buffer A where 3.2 mL fractions were collected, removing any unbound non-target proteins. To remove the bound enzyme, a 0–2 M KCl gradient with a total volume of 30 mL was applied to the column, and 1.6 mL fractions were collected. To assay subsequent enzyme activity, 10 µL from each fraction was used and fractions with the highest PK activity were pooled. The salt in the sample was also removed by passage through Sephadex G-25 in the same manner described for removing PEP from the red muscle samples. Following this procedure, the sample was then reapplied to a new CM^−^ Sephadex column (1 × 4 cm) that had been equilibrated with buffer A. After loading the sample, the column was washed with 30 ml of buffer A before eluting PK with a 0–15 mM PEP gradient while collecting 1.6 ml fractions. To assay activity, 10 µL aliquots from each fraction were used, and fractions that contained active PK activity were pooled together. As described previously, PEP was removed from pooled sample using G-25 Sephadex that was previously equilibrated with buffer B (pH 7.0, 20 mM MOPS, 2 mM EDTA, 2 mM EGTA, 15 mM β-glycerophosphate, 15 mM β-mercaptoethanol, and 10% v/v glycerol). The enzyme sample was then held at 4 °C until further use.

Pooled samples were assayed following each step to determine activity in both tissues for control and anoxic conditions. Protein concentrations were evaluated using the Bradford method (Bradford, 1976) and the Bio-Rad Protein Assay dye reagent with absorbance at 595 nm and using serial dilutions of bovine serum albumin as the standard according to the manufacturer’s instructions.

### Pyruvate kinase enzyme assay and kinetics

The activity of PK was measured as the rate of production of pyruvate from PEP in a coupled enzyme reaction with lactate dehydrogenase that measured the rate of NADH consumption with bovine heart lactate dehydrogenase (CAS 9001-60-9, Sigma-Aldrich). This was performed by measuring the absorbance at 340 nm using a BioTek spectrophotometer on a 96-well microplate. The optimal assay conditions for red muscle PK in the forward PEP consuming direction were 37.5 mM Tris pH 7.2, 7.45 mM KCl, 0.15 mM NADH, 0.5 mM PEP, 0.5 mM ADP, 5.5 mM Mg^++^, 1 U of LDH and 10 µL of enriched PK enzyme in a 200 µL reaction volume. The optimal conditions for liver PK in the forward direction were 36.25 mM Tris pH 7.2, 7.25 mM KCl, 0.15 mM NADH, 1 mM PEP, 1 mM ADP, 5 mM Mg^++^, 1 U of LDH, and 10 µL of enriched enzyme. Assays were typically initiated through the addition of PEP. As PK has an extremely high and negative ΔG only the activity in the forward direction was measured. All assays were run at room temperature (22 °C). *K*_*m*_ values for PEP and ADP and *I*_50_ values for KCl and urea were determined at constant, saturating co-substrate concentrations, as above. To determine the effect of pH on the *K*_*m*_ of PEP, all assay concentrations were the same as above, except 36.25 mM potassium phosphate at pH 6.6 was substituted for Tris. *I*_50_ values for ATP, L-alanine, and lactate and the *K*_*a*_ values for FBP and aspartate were determined at constant, saturating levels of substrate. Kinetic data were analyzed using the Kinetics v.3.5.1 program (Brooks, 1992).

### Gel electrophoresis and Coomassie Blue staining

The enriched PK fractions were separated on 10% SDS-PAGE gels by electrophoresis as per Xiong & Storey (2012). In short, gels were run for 50 min at 180 V. Following separation of the proteins, gels were stained for 30 min with Coomassie Brilliant Blue. After this, the stain was removed and destaining solution (10% acetic acid, 15% methanol) was applied to the gel until definite bands appeared (∼60 min). Gels were then rinsed in distilled water before being visualized using a Chemi-Genius Bio-Imaging system with GeneSnap software (Syngene, Frederick, MD).

### Western blotting

For both the red muscle and liver tissue, PTMs were assessed via immunoblotting. The protocol used for western blotting was as in Abboud & Storey (2013). For liver PK, samples were concentrated before use by ∼10-fold in 10 mL 10 kDa cut off Centricons (Millipore) via centrifugation at 7,720× g for 20 min. For control and anoxic liver and muscle PK, aliquots of the enriched protein were loaded onto 10% SDS-PAGE gels, in addition to pre-stained molecular weight standards (Cat# PM005-0500, FroggaBio, Toronto, Canada) and PK standard (Cat# 9001-59-6, Sigma Aldrich, St. Louis, Missouri, USA). Following electrophoresis, proteins were electroblotted onto polyvinylidiene difluoride (PVDF) membranes (Millipore) by wet transfer. Membranes were blocked with 1% skim milk powder w/v diluted in TBST for 30 min before being probed with primary antibodies overnight at 4 °C. The membranes were probed with rabbit anti-phospho-serine (Cat# 618100, Invitrogen, Carlsbad, CA, USA), rabbit anti-phospho-threonine (Cat# 718200, Invitrogen, Carlsbad, CA, USA), mouse anti-phospho-tyrosine (Cat# 615800, Invitrogen, Carlsbad, CA, USA), rabbit anti-methylated lysine (SPC-158F, StressMarq, Biosciences Inc., Victoria, BC, Canada), or rabbit muscle anti-pyruvate kinase (GTX107977, GeneTex, Irvine, CA, USA) primary antibodies, all diluted 1:1000 in TBST. After removal of the primary antibody, membranes were washed and probed with their respective secondary antibodies, either goat anti-rabbit IgG horseradish peroxidase-linked antibody (Cat# APA007P, Bioshop Canada Inc., Burlington, ON, Canada) or anti-mouse IgG-peroxidase antibody (Cat# APA005P.2, Bioshop Canada Inc., Burlington, ON, Canada), for 45 min in a 1:8,000 (v/v) dilution in TBST. Membranes were washed for 3 × 5 min and then developed using enhanced chemiluminescence and subsequent visualized protein bands were quantified using a GeneTools program (Syngene). To correct for the variations in sample loading immunoblotting band densities were normalized against the same band after it was re-stained with Coomassie blue, and then the control PK group was set to 1 as a reference.

### Bioinformatics and statistical analysis

Enzyme velocities were analyzed using a Microplate Analysis (MPA) program and kinetic parameters were derived using a nonlinear least squares regression computer program, Kinetics v.3.5.1 (Brooks, 1992; Brooks, 1994). RBioplot was employed for statistical analysis via a Student’s *t*-test (Zhang & Storey, 2016). Predicted methylation sites were determined using *Chrysemys picta belli* NCBI reference sequences for the PK muscle isoform XP_008165009.1 and the liver isoform XP_005280746.1. Both were inputted separately into the http://www.jci-bioinfo.cn/iMethyl-PseAAC webserver (Qiu et al., 2014). Phosphorylations on threonine residues were predicted using the same NCBI reference sequences with http://www.cbs.dtu.dk/services/NetPhos/ with a prediction score set to 0.75 (Blom, Gammeltoft & Brunak, 1999). Sequence alignments and % similarity between isozymes were determined using https://www.ebi.ac.uk/Tools/msa/clustalo/ (Li et al., 2015).

## Results

### Purification of red-ear slider red muscle and liver PK

**Figure 1:**
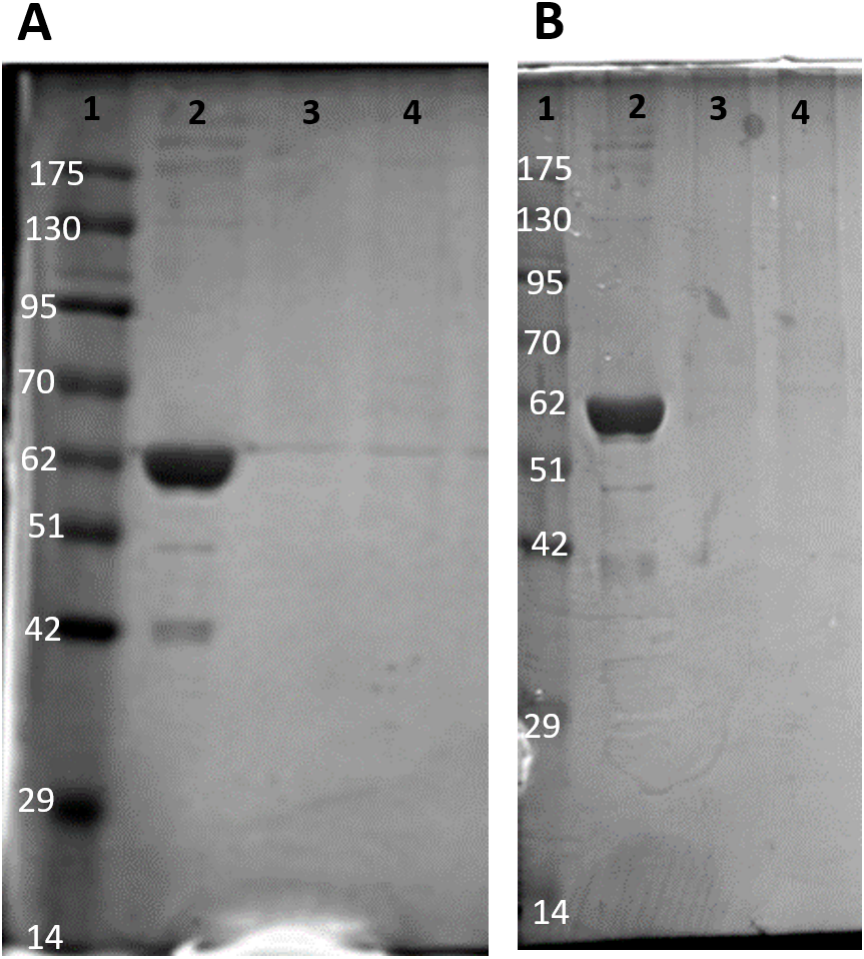
SDS-PAGE gel demonstrating a representative one step purification of red muscle PK from control (A) and 20-hr anoxic (B) turtles. Lanes are (1) 10–175 kDa protein ladder, (2) diluted PK standard (Cat# 9001-59-6, Sigma Aldrich, St. Louis, MO, USA), (3–4) eluate from CM^−^ Sephadex with a PEP gradient.

Purification of PK from red muscle of *T. s. elegans* was achieved using CM^−^ Sephadex with elution via a gradient of 0-15 mM PEP (Table 1). The purification resulted in an 82.8- and 97.6-fold purification with an associated specific activity of 14.5 U/mg and 11.8 U/mg for control and anoxic tissue, respectively. The final yield was also noted as 18.2 and 25.2% for control and anoxic tissue, respectively. Purity of the preparations was determined via SDS-PAGE stained with Coomassie Blue for the control and anoxic states (Fig. 1). PK was enriched to homogeneity with a band of PK at a molecular weight of approximately 57 kDa as illustrated by the PK standard (lane B).

**Table 1:** Representative purifications of pyruvate kinase from control (Top) and 20-hr anoxic (Bottom) *T. s. elegans* red muscle tissue.

Step	Activity (U)	Total protein (mg)	Specific activity (U/mg)	Fold purification	Yield (%)
Crude	10.1	57.6	0.17	–	–
CM Sephadex (PEP)	1.83	0.13	14.5	82.8	18.2
Crude	8.29	21.4	0.39	–	–
CM Sephadex (PEP)	2.09	0.18	11.8	97.6	25.2

**Figure 2:**
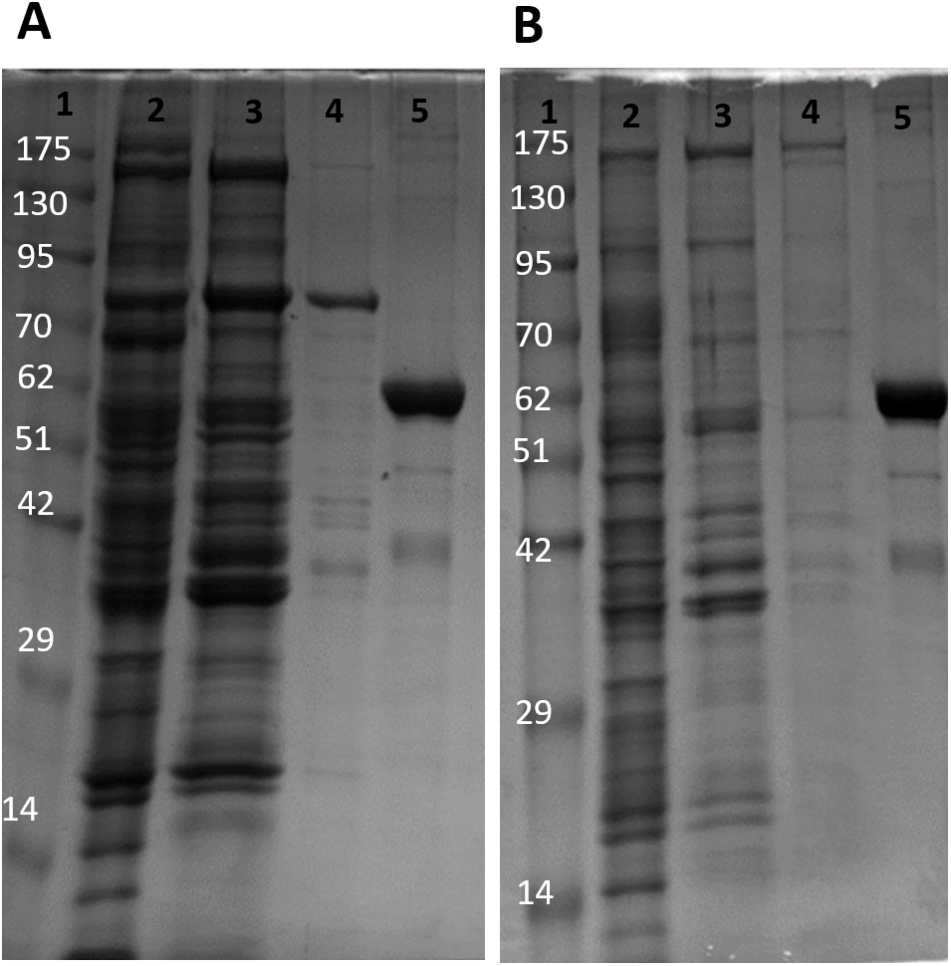
SDS-PAGE gel demonstrating a representative purification of liver PK from control (A) and 20-hr anoxic (B) turtles. Lanes are (1) 10–175 kDa protein ladder, (2) crude sample, (3) eluate from CM^−^ with KCl gradient column, (4) eluate from CM^−^ with PEP gradient column, and (5) diluted PK standard from rabbit muscle (Cat# 9001-59-6, Sigma Aldrich, St. Louis, MO, USA).

The partial purification of *T. s. elegans* liver PK was performed in two steps using CM^−^ Sephadex with gradients of KCl and PEP as elution methods (Table 2). Elution from the first CM^−^ column resulted in a 2.55-fold purification for control PK with an activity yield of 45.8%, and elution from the second CM^−^ column resulted in a final 7.30-fold purification and activity yield of 29.8% (Table 2). Similarly, anoxic PK eluted from CM^−^ using KCl had an initial fold purification of 1.93 and activity yield of 32.2%. When eluted from CM^−^ with PEP as a second step, anoxic PK had a fold purification of 4.16 and activity yield of 11.3 % (Table 2). The final specific activities achieved for control and anoxic PK were 1.32 U/mg and 0.49 U/mg respectively. An SDS-PAGE gel stained with Coomassie Blue was used to assess the purification process for both control and anoxic PK from liver (Fig. 2). Multiple bands were seen after the last step, with a band of liver PK at a molecular weight of 57 kDa.

**Table 2:** Representative purifications of pyruvate kinase from control (Top) and 20-hr anoxic (Bottom) *T. s. elegans* liver tissue.

Step	Activity (U)	Total protein (mg)	Specific activity (U/mg)	Fold purification	Yield (%)
Crude	2.52	13.9	0.18	–	–
CM Sephadex (KCl)	1.15	2.49	0.46	2.55	45.8
CM Sephadex (PEP)	0.75	0.57	1.32	7.30	29.8
Crude	1.31	11.1	0.12	–	–
CM Sephadex (KCl)	0.42	1.85	0.23	1.93	32.2
CM Sephadex (PEP)	0.15	0.30	0.49	4.16	11.3

**Figure 3:**
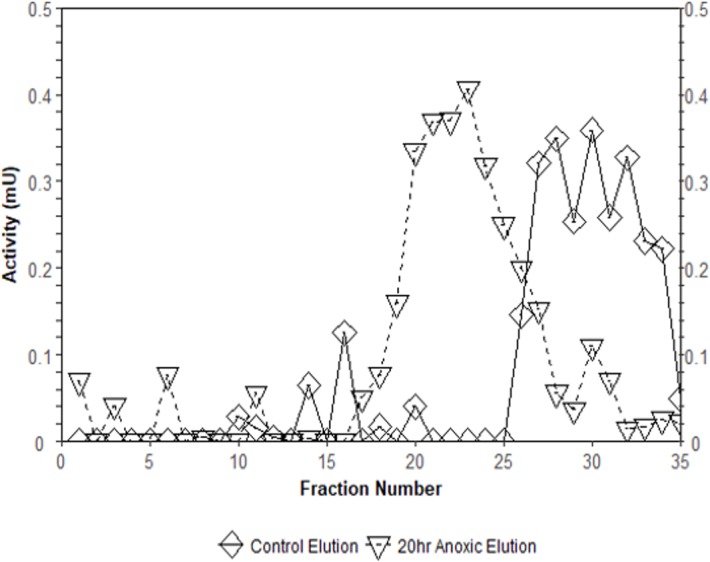
Comparison of elution profiles from CM^−^ Sephadex between control and 20-hr anoxia PK in *T. s. elegans* liver. Enzyme samples were bound to CM^−^ Sephadex beads at pH 6.0 and eluted using a 0–15 mM PEP gradient. Each point is the enzyme velocity from a single fraction tube assayed at room temperature with consistent substrate concentrations.

Interestingly, the purification of liver PK resulted in different elution profiles from the CM^−^ Sephadex column when comparing control and anoxic conditions (Fig. 3). Control PK began eluting from the column at an approximate concentration of 10.5 mM PEP whereas the anoxic PK counterpart began eluting at an approximate concentration of 3.2 mM PEP.

### Kinetic parameters of PK in control and anoxic red muscle PK

Kinetic parameters of muscle PK were evaluated for aerobic control and anoxic states (Table 3). Michaelis–Menten constants (*K*_*m*_) for both PK substrates, ADP and PEP, were determined (pH 7.2, 22°C). *K*_*m*_ PEP increased significantly from the control (0.011 ± 0.001 mM) to the 20-hr anoxic (0.061 ± 0.004 mM) condition, indicating a decrease in PEP affinity under anoxia (*p* = 6.41^−05^, Table 3). Similarly, when *K*_*m*_ was evaluated at pH 6.6 (22°C), the *K*_*m*_ doubled under anoxic conditions resulting in a significant decrease in substrate affinity between the control (0.029 ± 0.004 mM) to the anoxic (.057 ± .008 mM) state (*p* = 0.021). Finally, PK exhibited a decrease in affinity for ADP as demonstrated by the significant increase in *K*_*m*_ between the control (0.20 ± 0.01 mM) and 20-hr anoxia (0.28 ± 0.01 mM) at pH 7.2 as shown in Table 3 (*p* = 0.0002).

**Table 3:** Kinetic parameters of purified red muscle pyruvate kinase from control and 20-hr anoxic *T. s. elegans*, *n* = 4.

Enzyme parameters	Control	20 h Anoxia
Forward reaction (PEP to pyruvate)		
*K*_*m*_, ADP, pH 7.2 (mM)	0.20 ± 0.01	0.28 ± 0.01^a^
*K*_*m*_, PEP, pH 7.2 (mM)	0.011 ± 0.001	0.061±0.004^a^
*K*_*m*_, PEP, pH 6.6 (mM)	0.029 ± 0.004	.057 ± .008^a^
*I*_50_, L-alanine, pH 7.2 (mM)	N/A	N/A
*I*_50_, ATP, pH 7.2 (mM)	11.6 ± 0.7	7.9 ± 0.1^a^
*I*_50_, ATP + FBP, pH 7.2 (mM)	N/A	N/A
*I*_50_, lactate, pH 7.2 (mM)	34.4 ± 2.4	177.6 ± 4.8^a^
*I*_50_, KCl, pH 7.2 (M)	0.054 ± 0.02	0.051 ± 0.02
*I*_50_, urea, pH 7.2 (M)	1.91 ± 0.09	1.46 ± 0.03^a^

**Notes.**

a denotes significance from control (*p* < 0.05).

Numerous putative metabolite inhibitors were assessed to determine their effects on muscle PK (Table 3). There was a significant decrease in *I*_50_ for ATP from the control (11.6 ± 0.7 mM) to the anoxic (7.9 ± 0.1 mM) (*p* = 0.0021) state. Interestingly, neither L-aspartate nor FBP had an influence on muscle PK in terms of activation. However, when determining the *I*_50_ for ATP in the presence of FBP it was determined that ATP had no discernable inhibitory effect; i.e., FBP (1.5 mM) overrode the inhibitory effect of ATP. L-Alanine did not show any discernable effect on control or anoxic muscle PK within physiological concentrations of alanine (up to 10 mM). The *I*_50_ for lactate increased by four-fold from 34.4 ± 2.4 mM to 177.6 ± 4.8 mM for the control and anoxic conditions, respectively (*p* = 1.43^−08^, Table 3). Finally, stability was evaluated using denaturants including urea and KCl. The *I*_50_ values for KCl under the control and anoxic conditions were 0.054 ± .02 M and 0.051 ± 0.02 M, respectively (*p* = 0.2) whereas those for urea were 1.91 ± 0.09 M and 1.46 ± 0.03 M, respectively (*p* = 0.037, Table 3).

### Kinetic parameters of PK in control and anoxic liver PK

Kinetic changes in liver PK properties were also assessed for aerobic control versus anoxic states for both the specific substrates of PK and the effects of known activators and inhibitors (Table 4). At pH 7.2, (22 °C), the *K*_*m*_ values for PEP were significantly different between control and anoxic states with respective values of 0.31 ± 0.01 mM and 0.55 ± 0.01 mM (*p* = 7.52 × 10^−8^, Table 4). Hence, under anoxic conditions, the *K*_*m*_ PEP was increased two-fold when compared to control values. Additionally, when the pH was reduced to 6.6, the *K*_*m*_ PEP decreased significantly for both control and anoxic PK when compared to their respective pH 7.2 values (*p* = 1.22 × 10^−5^, *p* = 8.24 × 10^−8^). However, anoxic PK at pH 6.6 yielded a *K*_*m*_ value of 0.092 ± 0.005 mM, a significant 1.8-fold increase from the measured control value of 0.051 ± 0.02 mM (*p* = 0.0001, Table 4). The second specific substrate, ADP, showed no significant change in *K*_*m*_ values between control and anoxic conditions with respective values of 0.12 ± 0.01 mM and 0.13 ± 0.004 mM (Table 4).

**Table 4:** Kinetic parameters of liver pyruvate kinase from control and 20-hr anoxic *T. s. elegans*, *n* = 4 − 6.

Enzyme parameters	Control	20 h Anoxia
Forward reaction (PEP to pyruvate)		
*K*_*m*_, ADP, pH 7.2 (mM)	0.12 ± 0.01	0.13 ± 0.004
*K*_*m*_, PEP, pH 7.2 (mM)	0.31 ± 0.01	0.55 ± 0.01^a^
*K*_*m*_, PEP, pH 6.6 (mM)	0.051 ± 0.02	0.092 ± 0.005^a^
*I*_50_, L-alanine, pH 7.2 (mM)	0.10 ± 0.01	0.01 ± 0.002^a^
*I*_50_, ATP, pH 7.2 (mM)	2.5 ± 0.2	2.7 ± 0.2
*I*_50_, ATP + FBP, pH 7.2 (mM)	2.0 ± 0.2	1.1 ± 0.2^a^^,^^b^
*I*_50_, lactate, pH 7.2 (mM)	187 ± 6	131 ± 3^a^
*I*_50_, KCl, pH 7.2 (M)	0.433 ± 0.009	0.41 ± 0.02
*I*_50_, urea, pH 7.2 (M)	1.41 ± 0.02	1.98 ± 0.03^a^
*K*_*a*_, L-aspartate, pH 7.2 (mM)	N/A	N/A
*K*_*a*_, FBP, pH 7.2 (mM)	0.029 ± 0.007	0.0032 ± 0.002^a^
FBP fold activation, pH 7.2	1.77 ± 0.06	1.98 ± 0.07

**Notes.**

a denotes significance from control (*p* < 0.05).

b denotes significance from ATP *I*_50_ counterpart within stress tissue (*p* < 0.05).

Activation of liver PK by aspartate or FBP was analyzed under saturating substrate concentrations. All values were obtained at pH 7.2 and 22°C. For FBP, anoxic PK showed a significantly lower *K*_*a*_ of 0.0032 ± 0.002 mM, a 90% decrease compared to the control value of 0.029 ± 0.007 mM (*p* = 4.22 × 10^−05^, Table 4). However, there was no significant difference for the fold activation of PK by FBP between control and anoxic conditions: values were 1.77 ±0.06 and 1.98 ± 0.07 mM, respectively. L-aspartate had no significant effect on the activity of PK between the concentrations of 0–20 mM.

The effects of inhibitors on the relative activities of control and anoxic liver PK were also determined (pH 7.2, 22°C). There were significant decreases in *I*_50_ values between control and anoxic PK for both alanine and lactate parameters. L-Alanine inhibition of anoxic liver PK (*I*_50_ = 0.01 ±  0.002 mM) exhibited an 86% decrease, within physiological concentrations of alanine (up to 2 mM), when compared to the control value (*I*_50_ = 0.10 ±  0.01 mM) (*p* = 0.002, Table 4). Comparatively, a 30% decrease in *I*_50_ values was observed for lactate when applied to anoxic PK, with the control lactate *I*_50_ being 187 ± 6 mM whereas anoxic PK showed a lactate *I*_50_ of 131 ± 3 mM (*p* = 4.22 × 10^−5^, Table 4). There was no significant difference in ATP inhibition of control versus anoxic PK yielding *I*_50_ values of 2.5 ± 0.2 mM and 2.7 ± 0.2 mM, respectively. However, when FBP was added to the assay at *K*_*a*_ activating concentrations, the *I*_50_ of ATP decreased significantly between control and anoxic states. In the presence of ATP, the anoxic PK had an ATP *I*_50_ value of 1.1 ± 0.2 mM, a 45% decrease from the control value (*p* = 0.04, Table 4). Finally, the *I*_50_ of urea for anoxic PK increased 1.4-fold from a control value of 1.41 ± 0.02 M to an anoxic value of 1.98 ± 0.07 M (*p* = 0.002), whereas the KCl *I*_50_ values for the enzyme exhibited no significant differences between control and anoxic PK states.

### Posttranslational modifications of PK from control and anoxic PK

**Figure 4:**
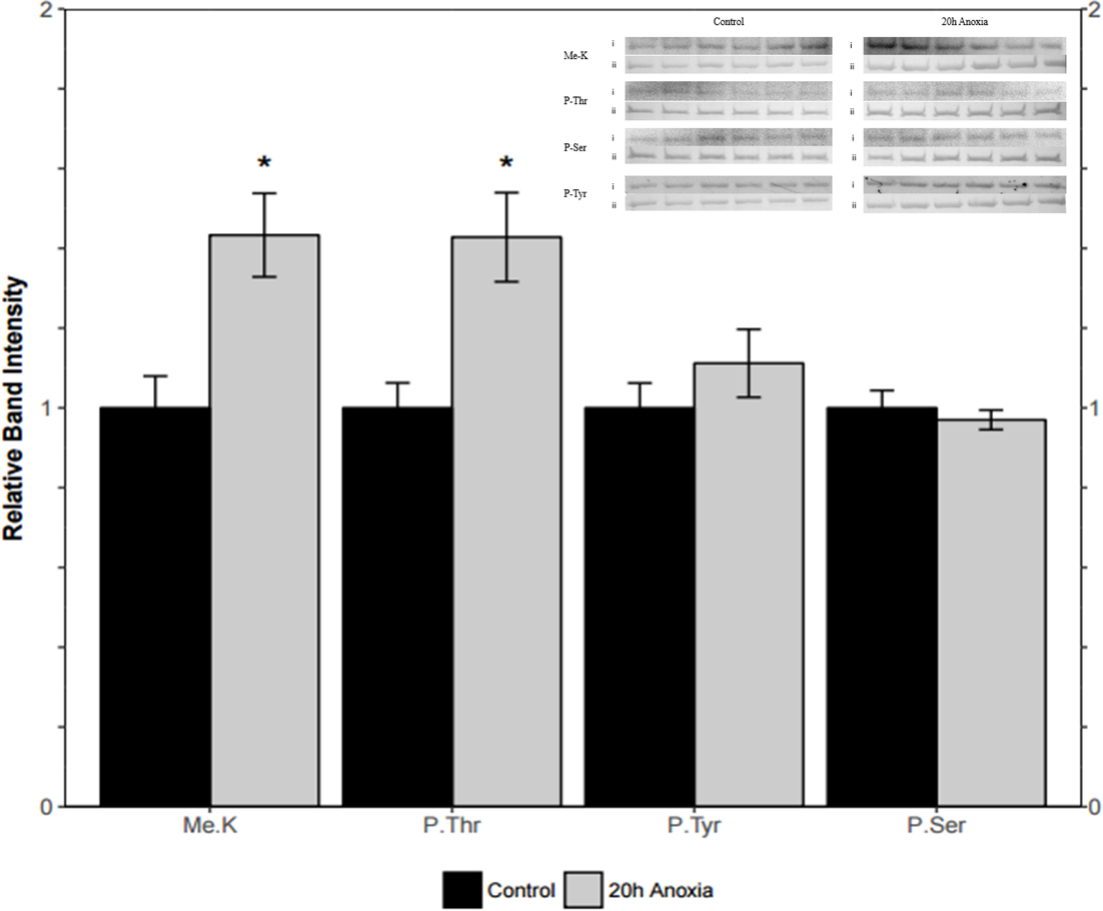
Quantification of post-translational modifications of partially purified control and 20-hr anoxic red muscle PK. Fold changes in phosphorylation and methylation of control and anoxic PK are shown. In each lane, immunoblotting band density was normalized against the same band after it was re-stained with Coomassie blue, and then the control PK group was set to 1 as a reference. The histogram reports values as a mean ± SEM for *n* = 6 independent samples. Representative bands from the (i) ECL and (ii) Coomassie blue stained membranes are shown above the histogram for each post-translational modification. Asterisks indicate significant differences from the corresponding control PK levels determined from a Student’s *t*-test, *p* < 0.05.

Immunoblotting was utilized to assess differences in protein post-translational modifications between control and anoxic PK in turtles. Anoxic red muscle PK showed a 42% increase in threonine phosphorylation relative to the control condition as shown in Fig. 4 (*p* = 0.02). Furthermore, lysine methylation of muscle PK increased significantly by 43% (*p* = 0.01). However, there were no significant differences in neither tyrosine nor serine residue phosphorylation between the control and anoxic states (Fig. 4).

**Figure 5:**
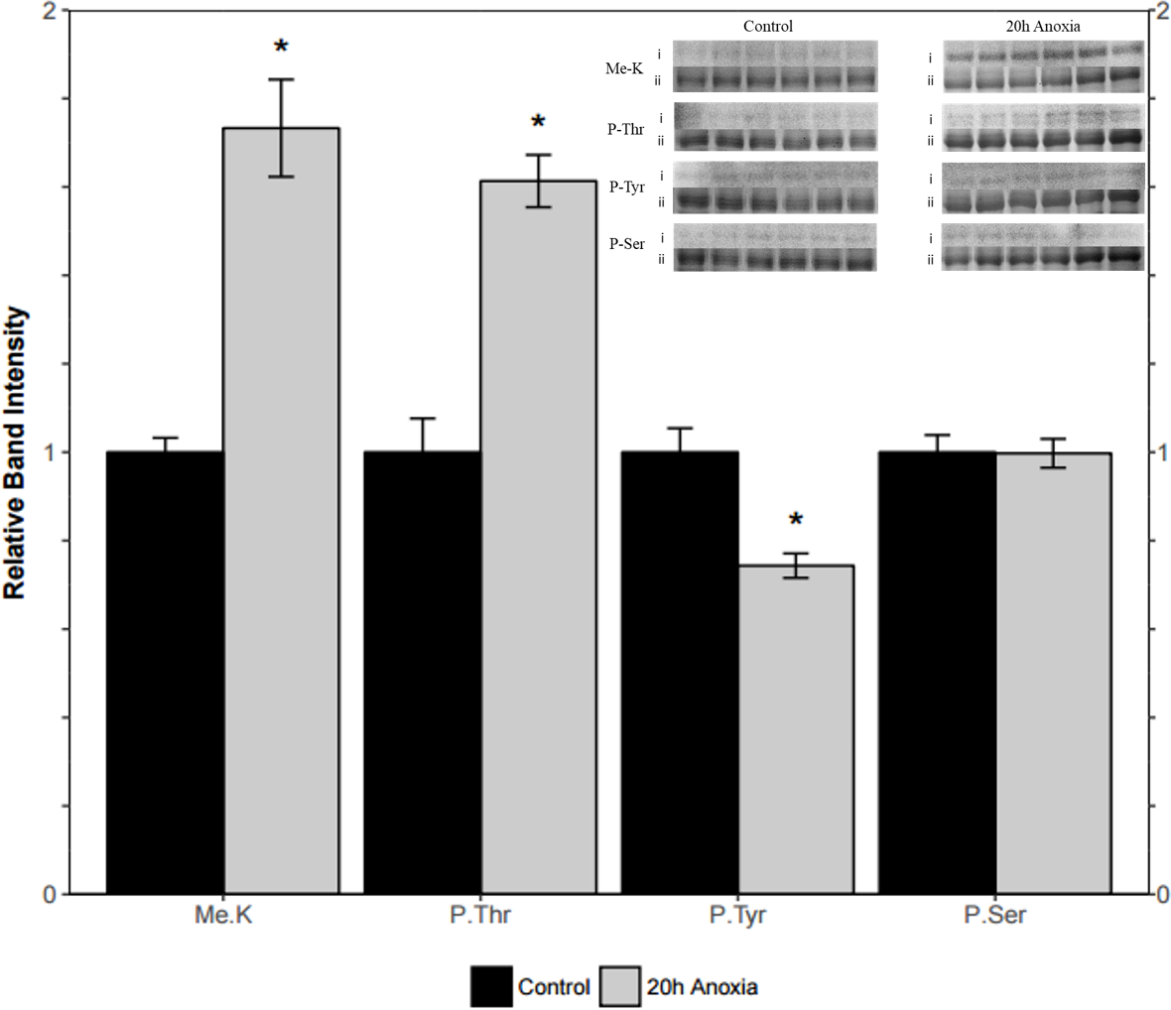
Quantification of post-translational modifications of partially purified control and 20-hr anoxic liver PK. Fold changes in phosphorylation and methylation of control and anoxic PK are shown. In each lane, immunoblotting band density was normalized against the corresponding band stained with Coomassie blue, and the control PK group was set to 1 as a reference. The histogram reports values as a mean ± SEM for *n* = 6 independent samples. Representative bands from the (i) ECL and (ii) Coomassie blue stained membranes are shown above the histogram for each post-translational modification. Asterisks indicate significant differences from the corresponding control PK levels determined from a Student’s *t*-test, *p* < 0.05.

Liver PK showed similar results where threonine phosphorylation levels increased significantly by 60% from the control to the anoxic state (*p* = 0.005, Fig. 5). Conversely, phosphorylation on tyrosine residues decreased by 26% for anoxic PK relative to control (*p* = 0.01, Fig. 5) whereas serine phosphorylation was unchanged. Finally, lysine methylation increased dramatically in the anoxic state by 70% relative to the control condition (*p* = 0.002, Fig. 5). The identification of PK was achieved using a specific antibody to the enzyme, yielding a fluorescent band around 57 kDa, which corresponded to the PK standard previously described in ‘Purification of red-ear slider red muscle and liver PK’.

## Discussion

Periodic transitions between normoxic and hypoxic/anoxic states are a natural feature for winter survival by northern freshwater turtles including *T. s. elegans*. Not only do these turtles experience oxygen limitation daily when diving for food or escaping predation, but they also must endure prolonged periods of anoxia while overwintering in ice-locked lakes and ponds (Biggar, Groom & Storey, 2011). The ability of *T. s. elegans* to endure oxygen deprivation is therefore crucial to its survival. Anaerobic glycolysis is the main energy provider for turtles throughout the anoxic period (Lutz, Nilsson & Prentice, 2003; Storey & Storey, 2004; Storey, 2007). This use of anaerobic carbohydrate catabolism is supported by adaptations of the turtle to buffer and tolerate the buildup of lactate, the anaerobic glycolytic product (Storey, 2007). As such, research into the regulation of PK, that produces pyruvate for the lactate dehydrogenase reaction, may help to provide insight into the metabolic reorganization of turtles during periods of anaerobiosis.

PK was enriched to homogeneity from the red muscle and partially enriched from liver tissue of *T. s. elegans*. The identity of PK was confirmed by purifying the enzyme to a single band on an SDS-PAGE gel with a molecular weight comparable to the enzyme in other vertebrates and to a PK molecular weight standard obtained from rabbit muscle (Fig. 1). Since specific activity increased throughout both red muscle and liver PK purification schemes the protocols described here were successful in enriching the protein tissue homogenates for PK. Kinetic changes in PK between control and anoxic states were noted for both liver and muscle isozymes. In both tissues, significantly increased *K*_*m*_ PEP values were noted at both neutral (7.2), and more acidic (6.6) pH values (Tables 3 and 4), the lower pH being comparable to the lowest values recorded *in vivo* for anoxic turtles (Robin et al., 1981). These results indicate that PK substrate affinity and potentially *in vivo* enzyme activity are reduced in the anoxic state.

Additional analysis of liver PK showed that the *K*_*m*_ value of PEP for anoxic PK from liver at pH 6.6 was significantly reduced from its pH 7.2 counterpart. As turtles are known to accumulate lactate during prolonged periods of anoxia (causing a reduction in cell and plasma pH), an increase in substrate affinity is suggestive of continued enzyme functioning even in the face of acidity (Jackson, Crocker & Ultsch, 2000). However, it is also important to mention that inhibition by lactate was increased for anoxic liver PK as denoted by a reduced *I*_50_ value (Table 4) which indicates an inhibition of PK as lactate levels rise during prolonged anoxia. Taken together, changes in substrate affinity and susceptibility to allosteric inhibitors may be coordinately regulated to result in an overall decrease in PK function as part of anoxia-induced hypometabolism although still maintaining sufficient activity to support basal ATP production by anaerobic glycolysis.

Furthering this concept is the noted increased sensitivity to FBP activation by the anoxic PK form in liver (as determined by a significantly decreased *K*_*a*_ value) but significantly increased inhibition by ATP in the presence of FBP (as determined by a significantly decreased *I*_50_ value) (Table 4). Thus, even though anoxic PK can be activated by lower amounts of FBP, increased glycolytic action and production of ATP could dampen enzyme activity overall. Furthermore, L-alanine has been previously noted to act as an allosteric inhibitor of PK (Hochachka & Mustafa, 1972). Increased alanine inhibition was also noted for anoxic liver PK, contributing to the idea that metabolic end products tightly control glycolytic action during hypometabolism. Overall, it appears that PK activity is maintained during anoxia as it is required for a basal glycolytic ATP production. This maintenance appears to be through an increased substrate affinity and the feed-forward activation by FBP. Furthermore, PK activity receives negative feedback by lactate, ATP and L-alanine dampening enzyme activity. Overall the modification of PK activity during anoxia provides another piece of the mechanism that allows these turtles to meet energy demands via anaerobic glycolysis while simultaneously decreasing metabolic rate by 80–90% (Herbert & Jackson, 1985).

A reduction in PK activity during periods of anoxia has been well documented in literature. Notably, the dehydration tolerant frog *Xenopus laevis* has been shown to increase the affinity of PK for PEP when the enzyme is dephosphorylated, much like in the turtle (Dawson et al., In press). Furthermore, a change in PK activity was measured in adult mangrove snail (*Littorina scabra angulifera*) foot muscle in response to anoxia (Fields & Ellington, 1992). That study found that anoxic snails exhibited decreased enzyme activity compared to controls (13.6 versus 4.7 µmol min^−1^ g wet wt^−1^) with subsequent increases in *K*_*m*_ PEP values (Fields & Ellington, 1992). Decreased PK substrate affinity was also noted in *Onchidium tumidium*, an intertidal pulmonate, during bouts of anoxia (Ip et al., 1993). More recently, reduced PK activity was also noted in the jaw muscle of anoxic crabs (*Chasmagnathus granulatus*) fed a carbohydrate rich diet prior to entry into hypometabolism (Marqueze, Kucharski & De Silva, 2006). Decreased PK activity during anoxia has also been noted for *Busycotypus canaliculatum*, an anoxia-tolerant gastropod, with decreased PEP substrate affinity and increased L-alanine inhibition, these changes being paralleled across four tissue types: ventricle, foot, hepatopancreas, and kidney (Whitwam & Storey, 1990). Overall, the decrease in PK activity appears to be a common occurrence in organisms undergoing periods of anoxia.

**Figure 6:**
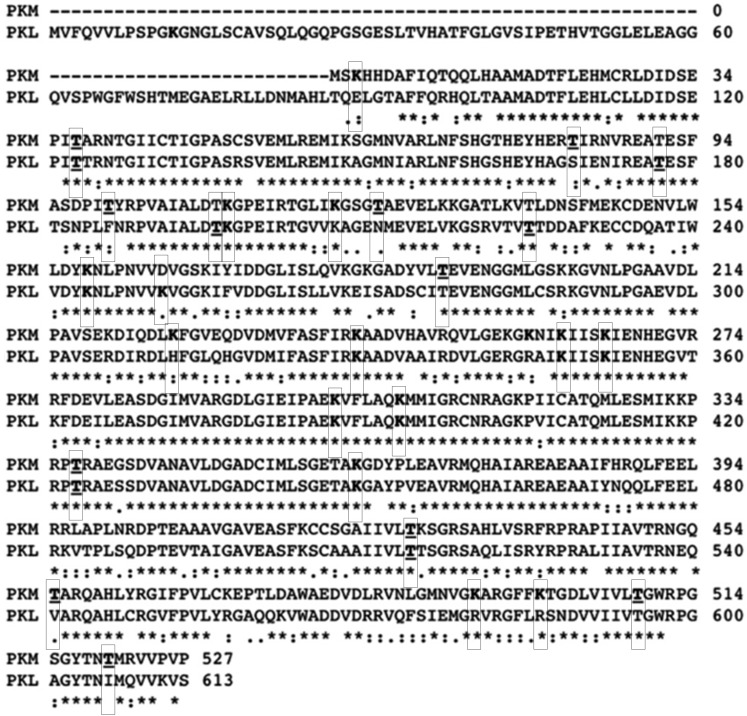
Muscle and liver pyruvate kinase sequence alignment. PK muscle (PKM) and liver (PKL) sequence from *Chrysemys picta belli* (NCBI reference sequence XP_008165009.1 and XP_005280746.1) aligned using Clustal Omega. Residues indicated with an ‘*’ (asterisk) are fully conserved, with a ‘:’ (colon) are groups of strongly similar properties as scoring >0.5 in the Gonnet PAM 250 matrix and with a ‘.’ (period) are groups of weakly similar properties scoring ≤0.5 and >0 in the Gonnet PAM 250 matrix. Dashes indicate a residue that is not present in both sequences. Clustal2.1 determined a 74% sequence identity between the isoforms. Predicted sites (boxed residues) from iMethyl-PseAAC for lysine methylation are in bold and for threonine phosphorylation from NetPhos3.1 are in bold and are underlined (Blom, Gammeltoft & Brunak, 1999; Qiu et al., 2014).

Similarly, in the red muscle of the turtle, a significant increase in *K*_*m*_ values for both ADP and PEP occurred (irrespective of pH) between normoxic and anoxic states (Table 3). As in the liver, this may lead to decreased PK activity during anoxia as part of metabolic rate depression. Furthermore, increased ATP inhibition was noted, also contributing to the notion that glycolytic rate undergoes a controlled overall reduction during prolonged oxygen deprivation. In contrast to liver PK, the lactate tolerance of red muscle PK greatly increased during anoxia (Table 3). This would suggest that sustained PK activity and glycolytic function occurs even in the face of a high accumulation of products, suggesting that red muscle PK is well suited for supporting glycolytic flux under anoxic conditions. A differential regulation of glycolytic enzymes according to tissue type has also been noted recently in the snail, *Otala lactea*, during estivation, another form of hypometabolism (MacLean et al., 2016). In snail foot muscle, LDH function was increased as supported by a reduced pyruvate *K*_*m*_ value. However, LDH function in the hepatopancreas decreased in the pyruvate-consuming direction during estivation (MacLean et al., 2016). These tissue-specific responses were proposed to be the result of a differential prioritization of metabolic action in each tissue, with foot muscle LDH favouring forward flux through glycolysis whereas the hepatopancreas enzyme may be primed for a role in gluconeogenesis (MacLean et al., 2016). A similar phenomenon may underlie PK control in turtle liver versus red muscle tissues. PK is known to exist in multiple tissue-specific isozyme forms; in mammals, for example, four PK isozymes exist (L, R, M_1_ and M_2_), each being differentially regulated (Jurica et al., 1998). The L form, found in the liver, is known to have multiple allosteric regulators, whereas the M_1_ form, found in adult skeletal muscle and brain is often regarded as a non-regulatory enzyme (Jurica et al., 1998). Sequence comparisons from a closely related turtle, *Chrysemys picta belli* revealed that turtle muscle and liver isozymes have a 74% sequence identity (Fig. 6). Therefore, differences in activity may be both due to isozyme differences along with differential regulation. Interestingly, it was noted that red muscle PK in the turtle was not activated by FBP or inhibited by L-alanine (Table 3). Decreased regulatory input on red muscle PK may be thus due to isozyme differences, and coordinately allow for differential regulation of PK in different tissues in the face of anoxia.

The question remains as to how kinetic changes in turtle PK are mechanistically brought about during anoxia. In both red muscle and liver, changes in PTMs of PK were noted during anoxia. Specifically, increased threonine phosphorylation was noted in both tissues, as was increased lysine methylation (Figs. 4 and 5). Interestingly, only liver tissue demonstrated a decrease in phosphorylation on tyrosine residues. This could provide insight into the nuanced kinetic alterations demonstrated by liver tissue such as the sensitivity to L-alanine that was not noted in the muscle isoform (Table 4 Fig. 5). Increased PK threonine phosphorylation resulting in suppression of PK has often been reported during anoxia and other forms of hypometabolism. In *B. canaliculatum*, for example, increased PK phosphorylation correlated with changes in enzyme properties that would suppress PK function (Whitwam & Storey, 1990). Furthermore, based on electrophoretic analysis of [^32^P] incorporation, it was suggested that O-phosphothreonine residue(s) were produced under anoxia (Plaxton & Storey, 1984). The increased phosphorylation of threonine residues on turtle PK during anoxia mirrors this result for the snail enzyme. Additionally, treatment of PK with an alkaline phosphatase allowed for the reversal of anoxia-induced PK suppression in *L. s. angulifera*, suggesting that changes in phosphorylation directly confer kinetic alterations of the enzyme (Fields & Ellington, 1992). By contrast, increases in PK activity were noted in other anoxia tolerant species such as the oyster (*Crassostrea virginica*) during bouts of hypometabolism, and this was accompanied by dephosphorylation of the enzyme (Greenway & Storey, 2000). Such results suggest phosphorylation control of PK activity in a uniform manner in response to anoxia or other forms of hypometabolism. The impact and ubiquitous presence of phosphorylation has lead recent reviews to consider the impact of other modifications. The phosphopreoteome of a human cancer cell line revealed that phosphorylated residues are preferentially located near modifiable lysines making crosstalk between the various PTMs an interesting avenue for study (Sharma et al., 2014). Here we demonstrate a coordinated increase in threonine phosphorylation and lysine methylation during anoxia (Figs. 4 and 5). Bioinformatic analysis of both pyruvate kinase sequences reveal two locations of threonine phosphorylation that are aligned in both isozymes while the isozymes share eight predicted methyl-lysine locations (Blom, Gammeltoft & Brunak, 1999; Qiu et al., 2014). This could indicate a more global regulation mechanism through PTMs on methyl-lysine while finer tuned regulation occurs with phosphorylation sites.

Differences in urea sensitivity were also noted between control and anoxic conditions for both liver and red muscle PK (Tables 3 and 4). Urea has been noted in previous studies as important in the osmoregulation of turtles during periods of dehydration and for tolerating increased salinity (Uchiyama et al., 2009; Hong et al., 2014). However, *T. s. elegans* undergoes anoxia at the bottom of lakes and is not faced with dehydration stress, so urea accumulation is negligible on a physiological level. However, urea is widely used as a chemical denaturant in studies of protein conformational stability and changes in sensitivity of a protein to urea can be interpreted as potential alterations in enzyme structural stability. Increased urea tolerance was noted for turtle liver PK (*I*_50_ values rose from 1.4 M in control to 2.0 M under anoxia), suggesting increased stability of the anoxic enzyme active site compared with the control enzyme. Increased PK stability would reduce protein turnover and therefore would be energetically advantageous under anaerobic conditions. Interestingly liver PK from these anoxic animals showed greater resistance to urea denaturation while the PK from red muscle was more sensitive to urea.

## Conclusions

Purification of PK from turtle red muscle and partial purification from liver allowed for analysis of enzyme kinetic parameters between normoxic and anoxic states. Overall, the data demonstrated a decrease in PK substrate affinities for PEP and ADP in the anoxic state, which suggests a depression of glycogen metabolism that likely contributes to metabolic rate depression in the turtle. In conclusion, the regulation of PK proves to be important in terms of controlling glycolytic flux in liver and muscle tissue. This study also suggests that threonine phosphorylation and lysine methylation may play key roles in altering substrate affinities thereby asserting posttranslational control over the pathway as shown in previous studies of metabolic responses to oxygen deprivation by anoxia tolerant species. This study provides new insights on how the freshwater turtles undergoes metabolic reorganization to cope with torpor.

##  Supplemental Information

10.7717/peerj.4918/supp-1 Supplemental Information 1 Supplementary raw data for enzyme kinetics

10.7717/peerj.4918/supp-2 Supplemental Information 2 Full coomassie blue stained PVDF membrane for liver threonine phosphorylation

10.7717/peerj.4918/supp-3 Supplemental Information 3 Full PVDF membrane for liver tyrosine phosphorylation

10.7717/peerj.4918/supp-4 Supplemental Information 4 Full coomassie blue stained PVDF membrane for liver lysine methylation

10.7717/peerj.4918/supp-5 Supplemental Information 5 Full stained PVDF membrane for liver serine phosphorylation

10.7717/peerj.4918/supp-6 Supplemental Information 6 Full coomassie blue stained PVDF membrane for liver serine phosphorylation

10.7717/peerj.4918/supp-7 Supplemental Information 7 Full PVDF membrane for liver threonine phosphorylation

10.7717/peerj.4918/supp-8 Supplemental Information 8 Full PVDF membrane for muscle serine phosphorylation

10.7717/peerj.4918/supp-9 Supplemental Information 9 Full coomassie blue stained PVDF membrane for muscle serine phosphorylation

10.7717/peerj.4918/supp-10 Supplemental Information 10 Full PVDF membrane for muscle lysine methylation

10.7717/peerj.4918/supp-11 Supplemental Information 11 Full coomassie blue stained PVDF membrane for muscle threonine phosphorylation

10.7717/peerj.4918/supp-12 Supplemental Information 12 Full coomassie blue stained PVDF membrane for liver lysine methylation

10.7717/peerj.4918/supp-13 Supplemental Information 13 Full coomassie blue stained PVDF membrane for muscle lysine methylation

10.7717/peerj.4918/supp-14 Supplemental Information 14 Full PVDF membrane for muscle tyrosine phosphorylation

10.7717/peerj.4918/supp-15 Supplemental Information 15 Full coomassie blue stained PVDF membrane for liver tyrosine phosphorylation

10.7717/peerj.4918/supp-16 Supplemental Information 16 Full PVDF membrane for muscle threonine phosphorylation

10.7717/peerj.4918/supp-17 Supplemental Information 17 Full coomassie blue stained PVDF membrane for muscle tyrosine phosphorylation

## Abbreviations

PEP: Phosphoenolpyruvate
PK: Pyruvate Kinase
PTMs: Posttranslational modifications
PMSF: Phenylmethylsulfonyl fluoride
SDS-PAGE: Sodium Dodecyl Sulfate Polyacrylamide Gel Electrophoresis
TBST: Tris-buffered saline with Tween
TBS: Tris-buffered saline
FBP: Fructose-1,6,-bisphosphate

## Additional Information and Declarations

### Data Availability

The following information was supplied regarding data availability: The raw data are provided in the Supplementary File.
