# Supplementary figures and images for: ­Characterization of pyruvate kinase from the anoxia tolerant turtle, Trachemys scripta elegans: a potential role for enzyme methylation during metabolic rate depression
Source: PeerJ. 2018 Jun 8;6:e4918. doi: 10.7717/peerj.4918 (PMC5995096; doi:10.7717/peerj.4918)

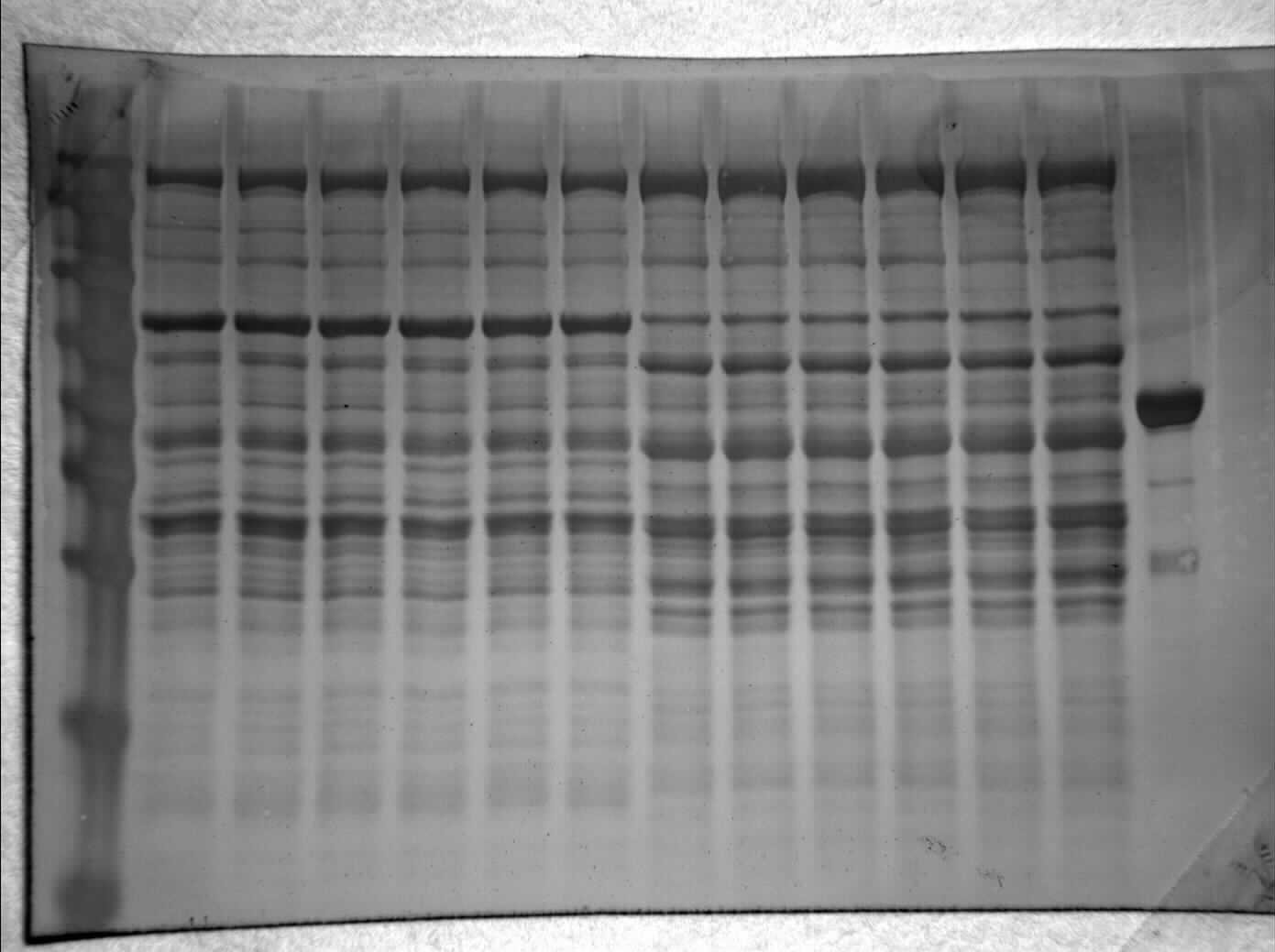

Supplement: Supplemental Information 2 [file peerj-06-4918-s002.jpg]

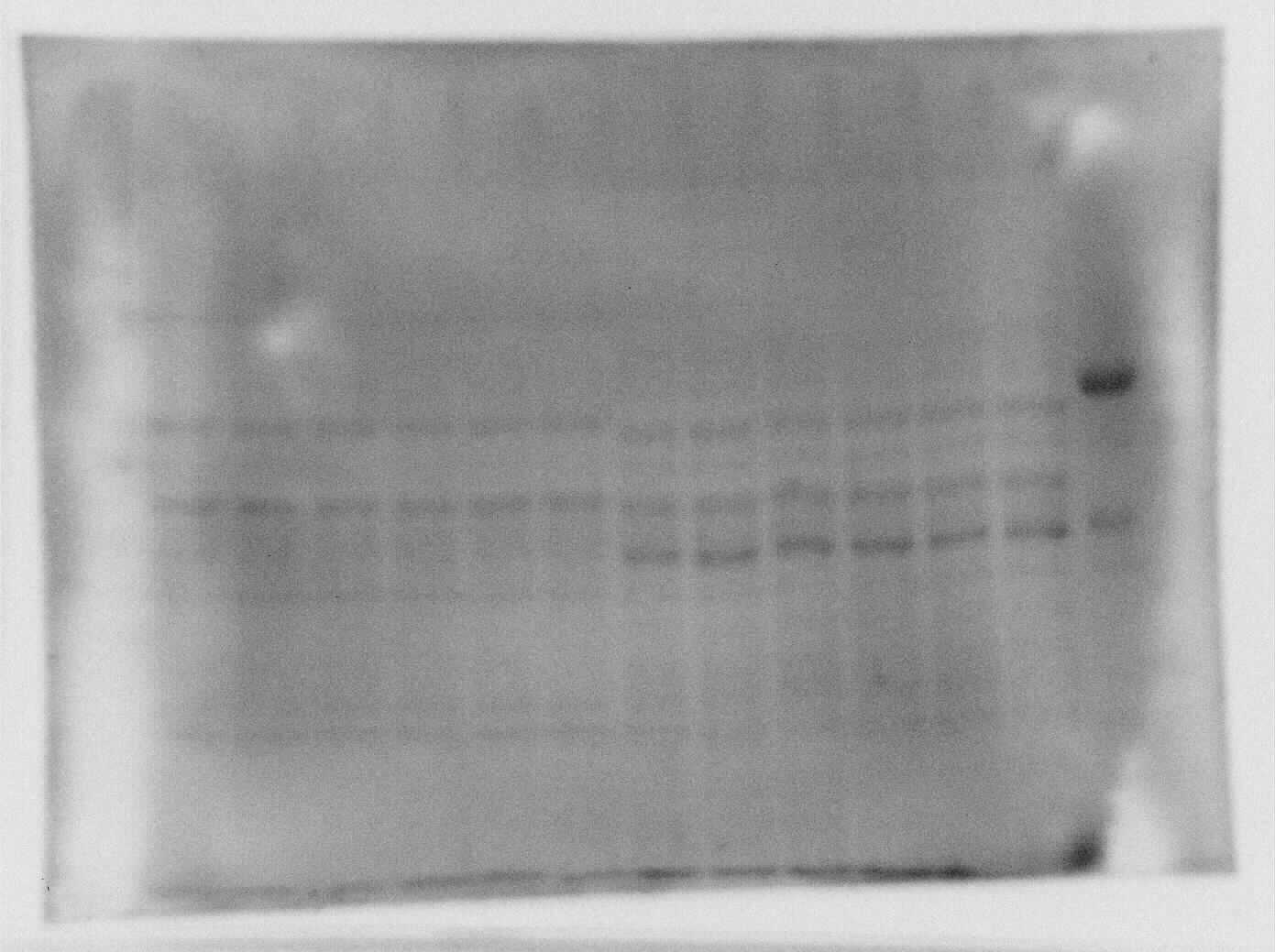

Supplement: Supplemental Information 3 [file peerj-06-4918-s003.jpg]

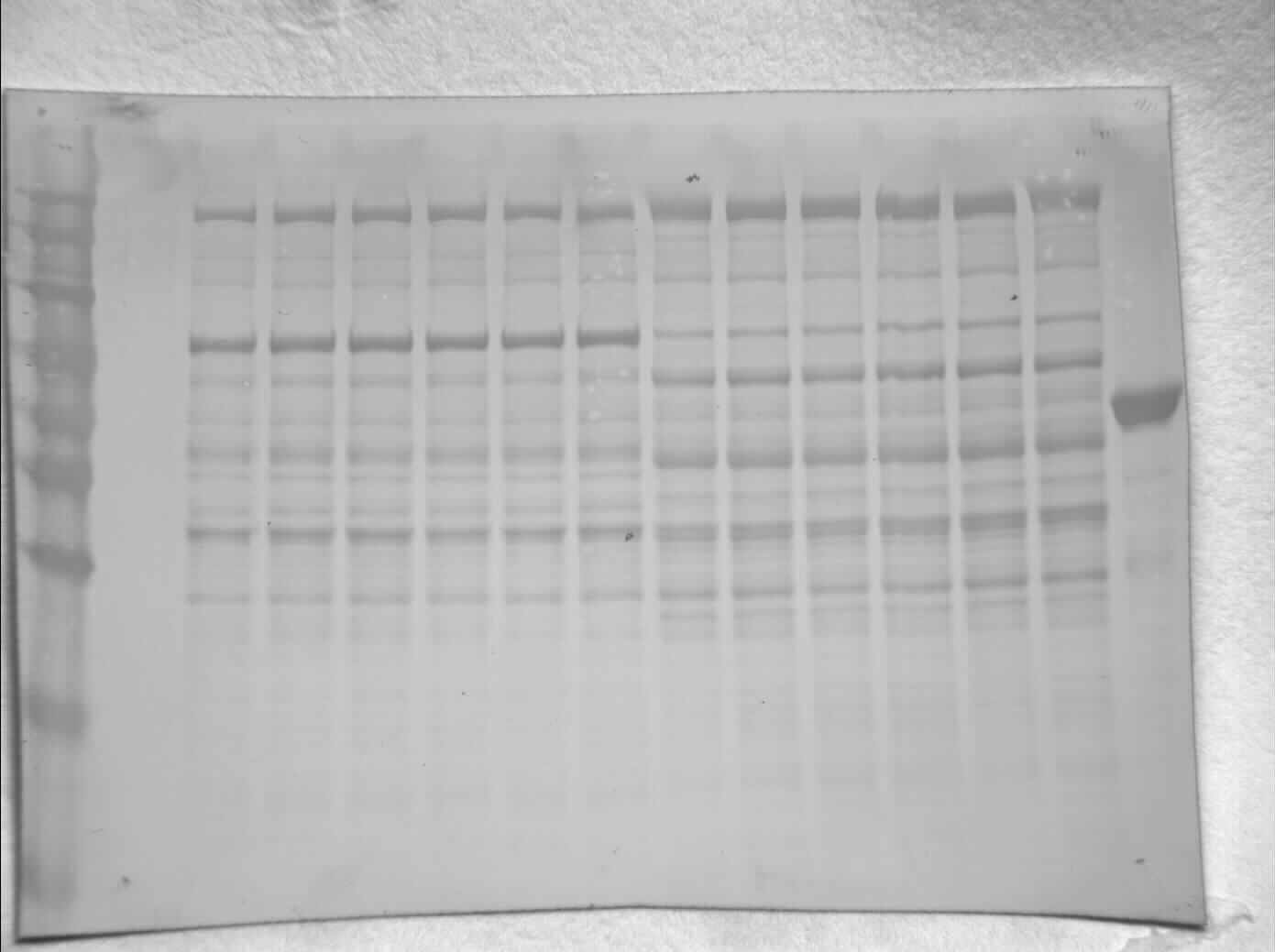

Supplement: Supplemental Information 4 [file peerj-06-4918-s004.jpg]

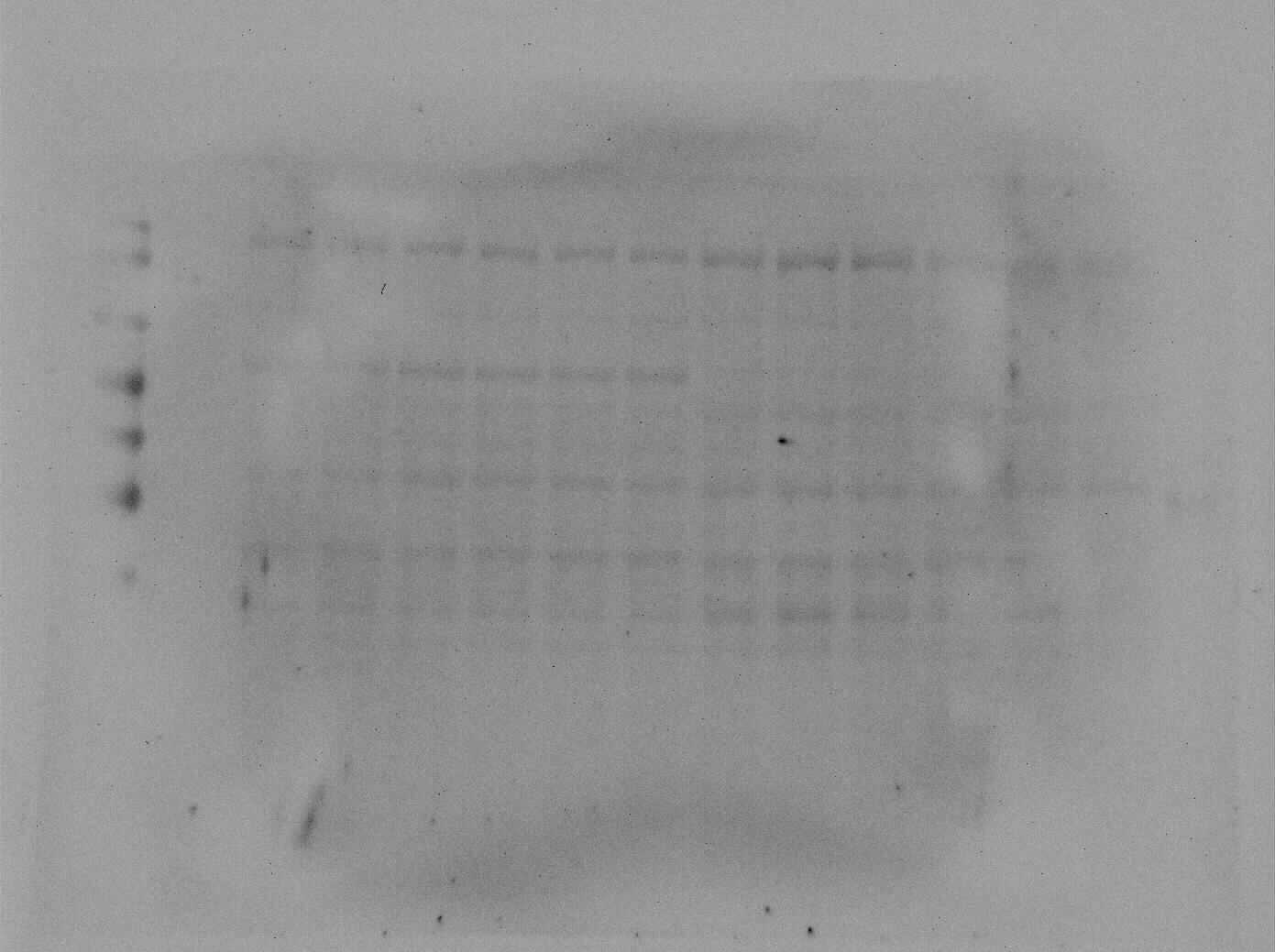

Supplement: Supplemental Information 5 [file peerj-06-4918-s005.jpg]

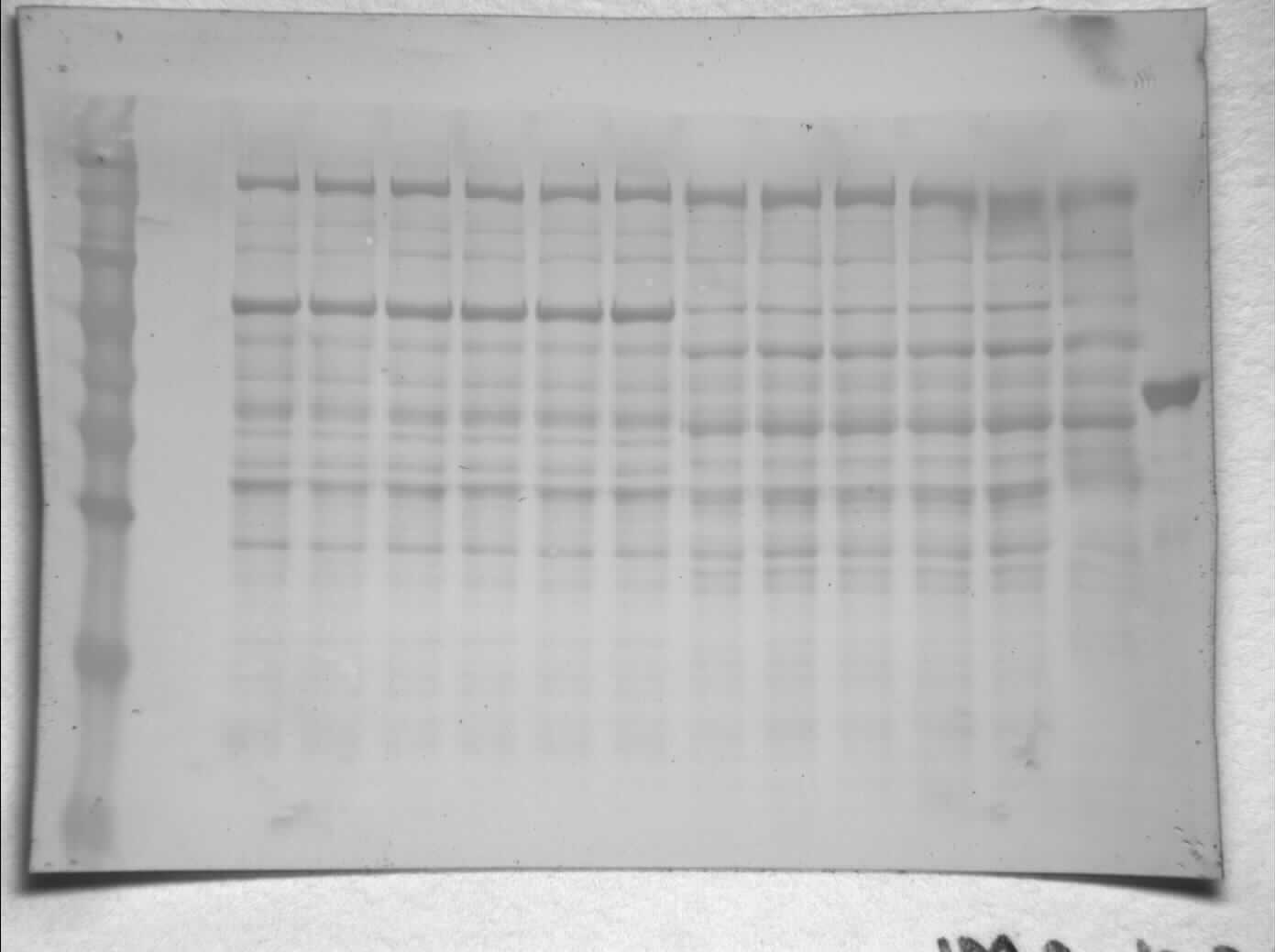

Supplement: Supplemental Information 6 [file peerj-06-4918-s006.jpg]

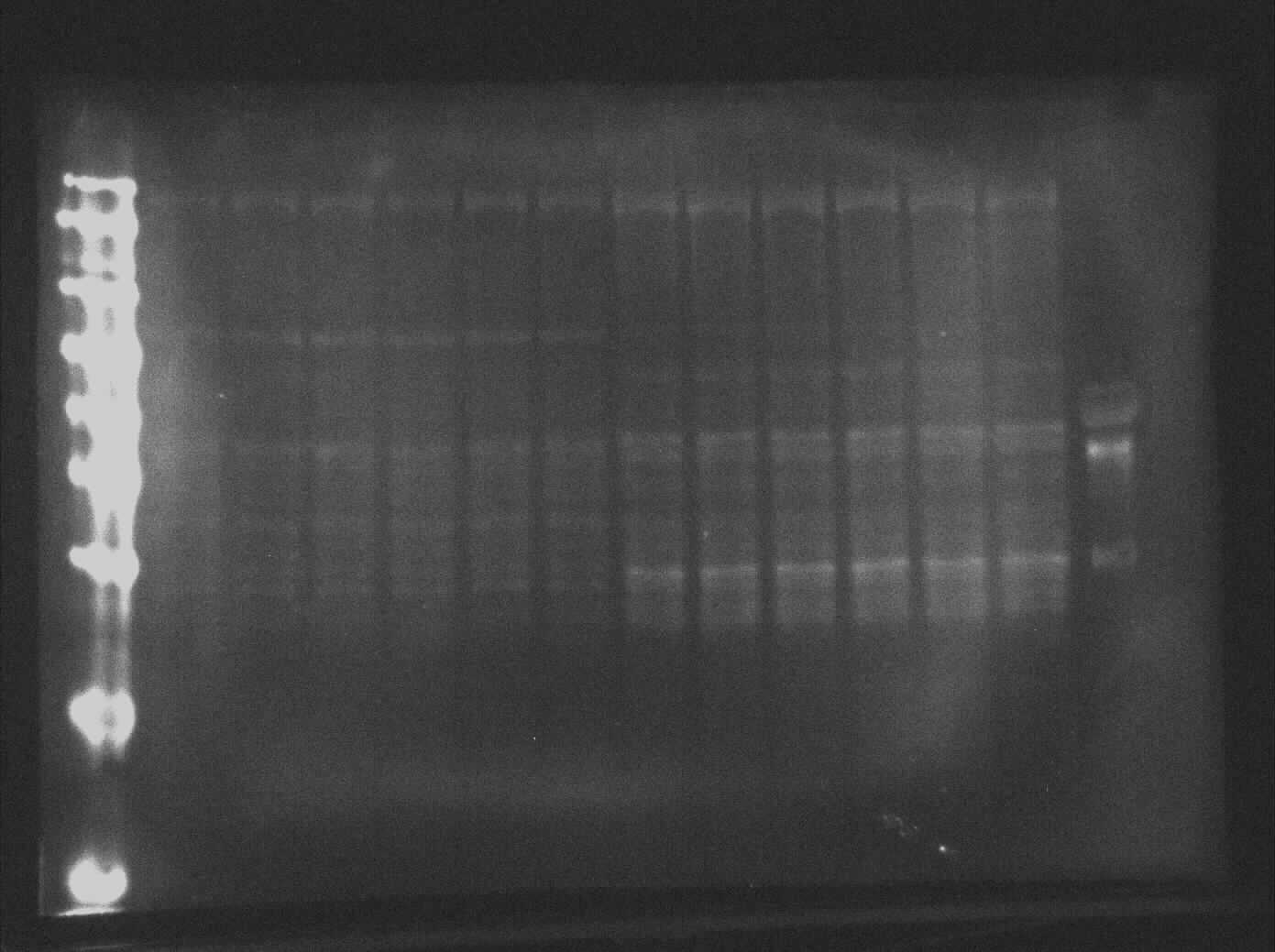

Supplement: Supplemental Information 7 [file peerj-06-4918-s007.jpg]

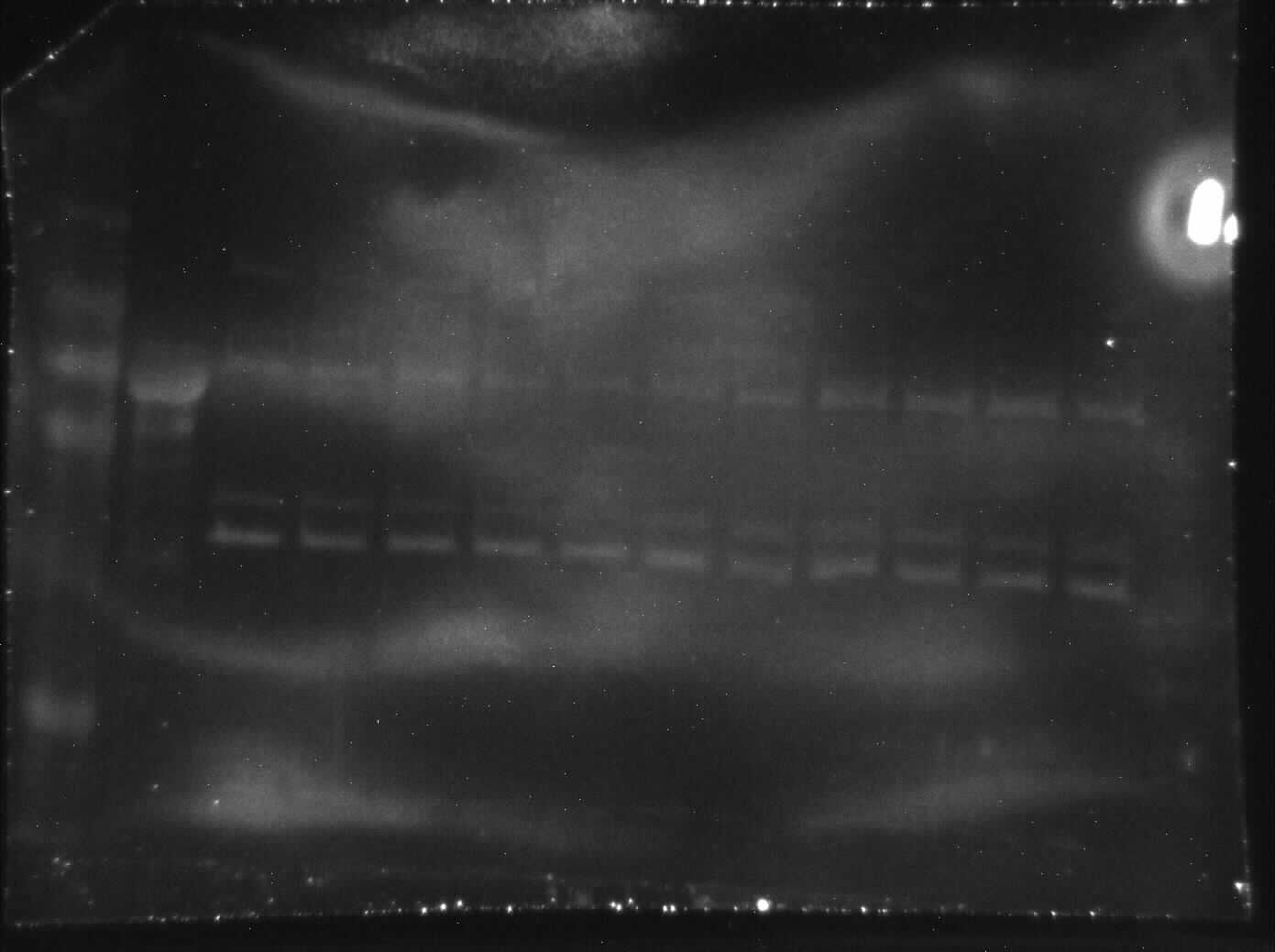

Supplement: Supplemental Information 8 [file peerj-06-4918-s008.jpg]

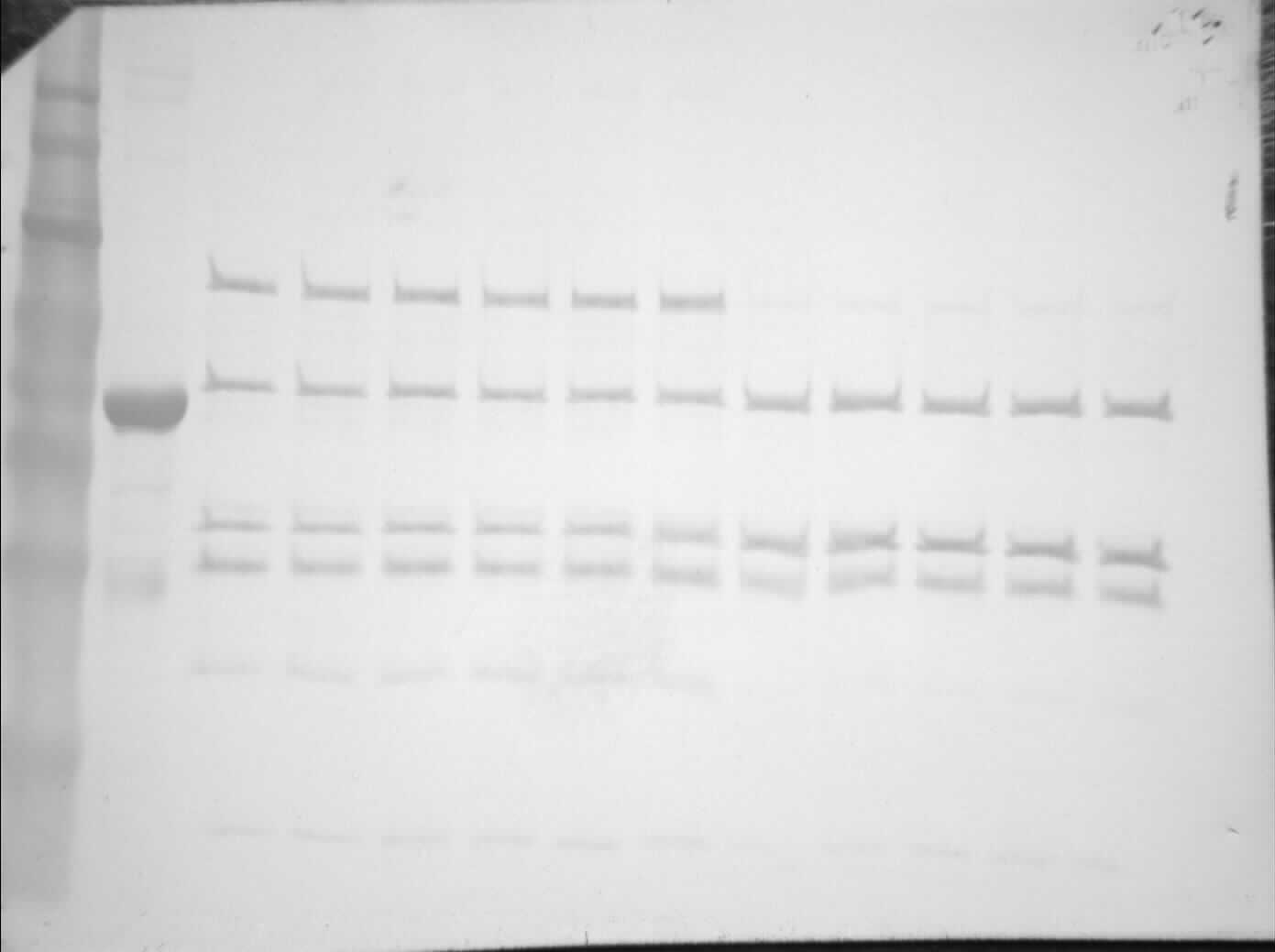

Supplement: Supplemental Information 9 [file peerj-06-4918-s009.jpg]

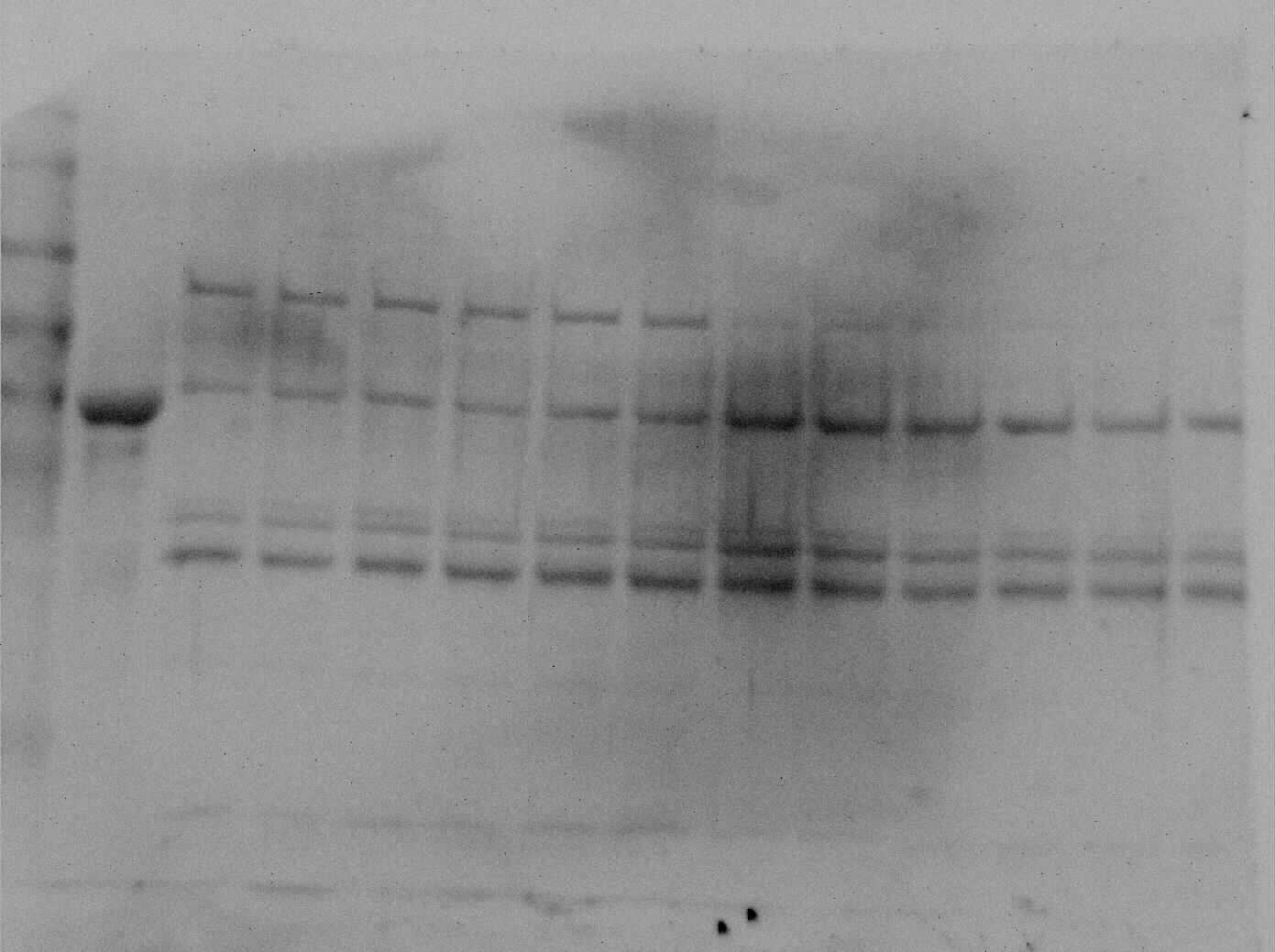

Supplement: Supplemental Information 10 [file peerj-06-4918-s010.jpg]

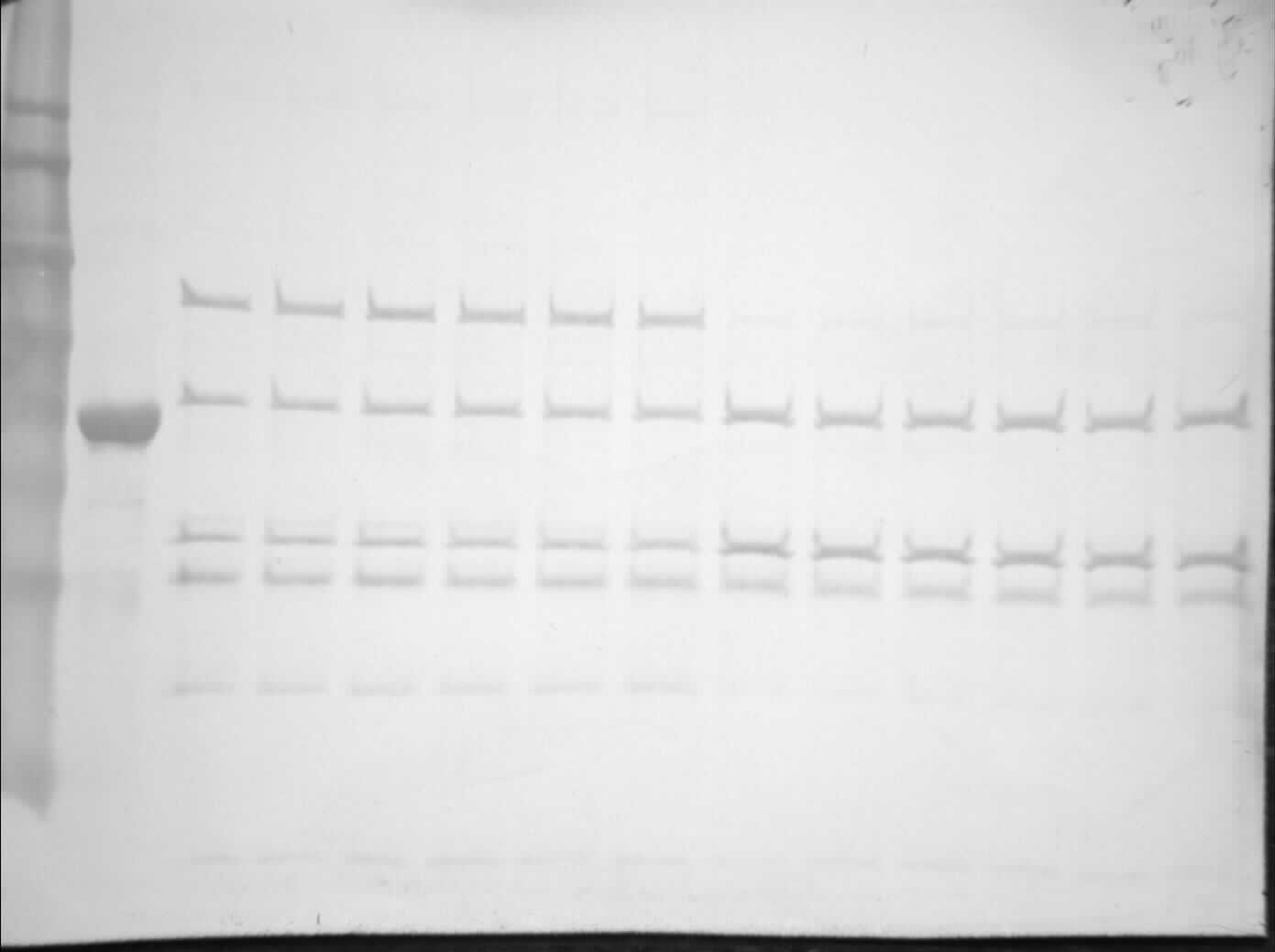

Supplement: Supplemental Information 11 [file peerj-06-4918-s011.jpg]

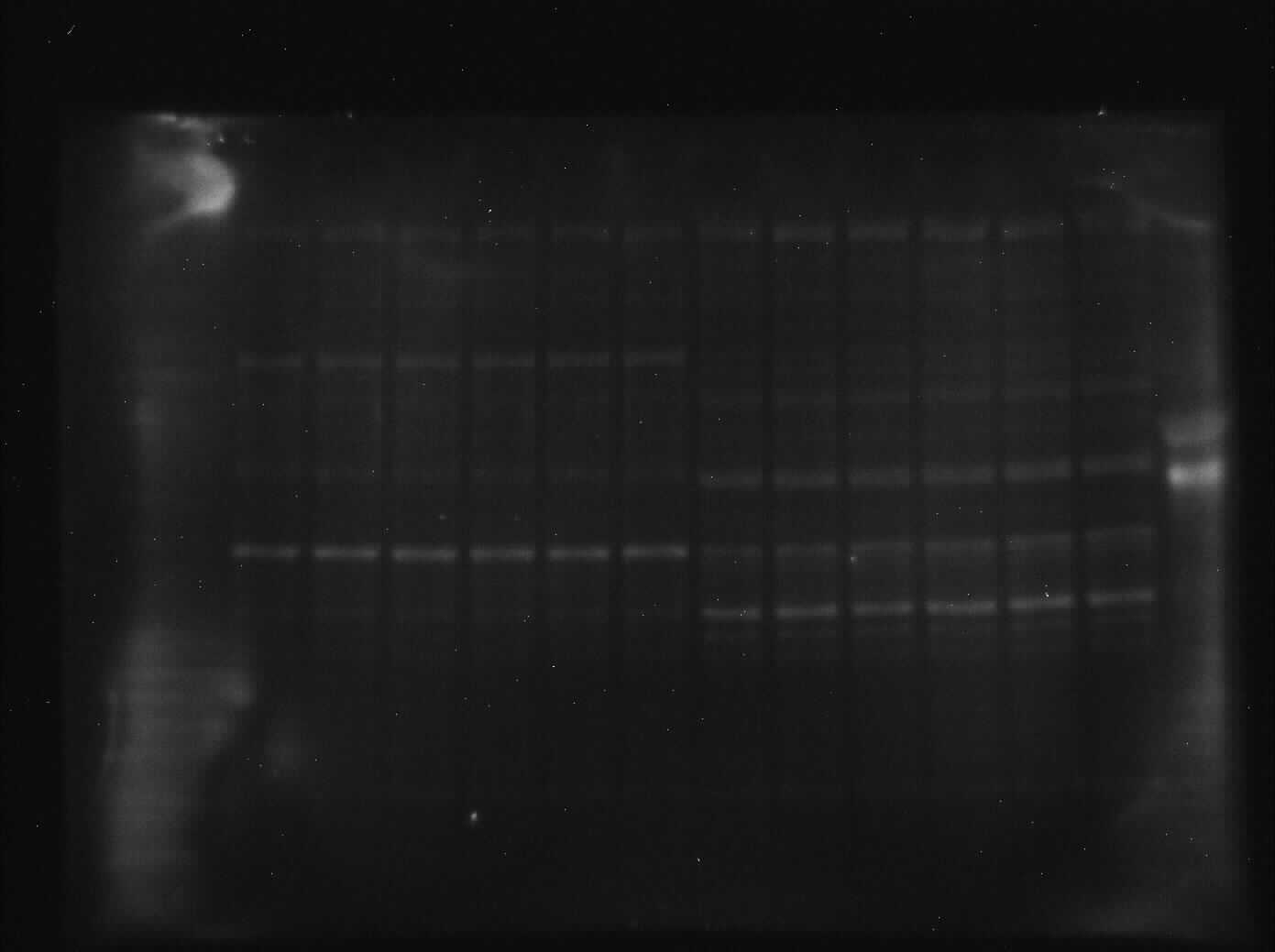

Supplement: Supplemental Information 12 [file peerj-06-4918-s012.jpg]

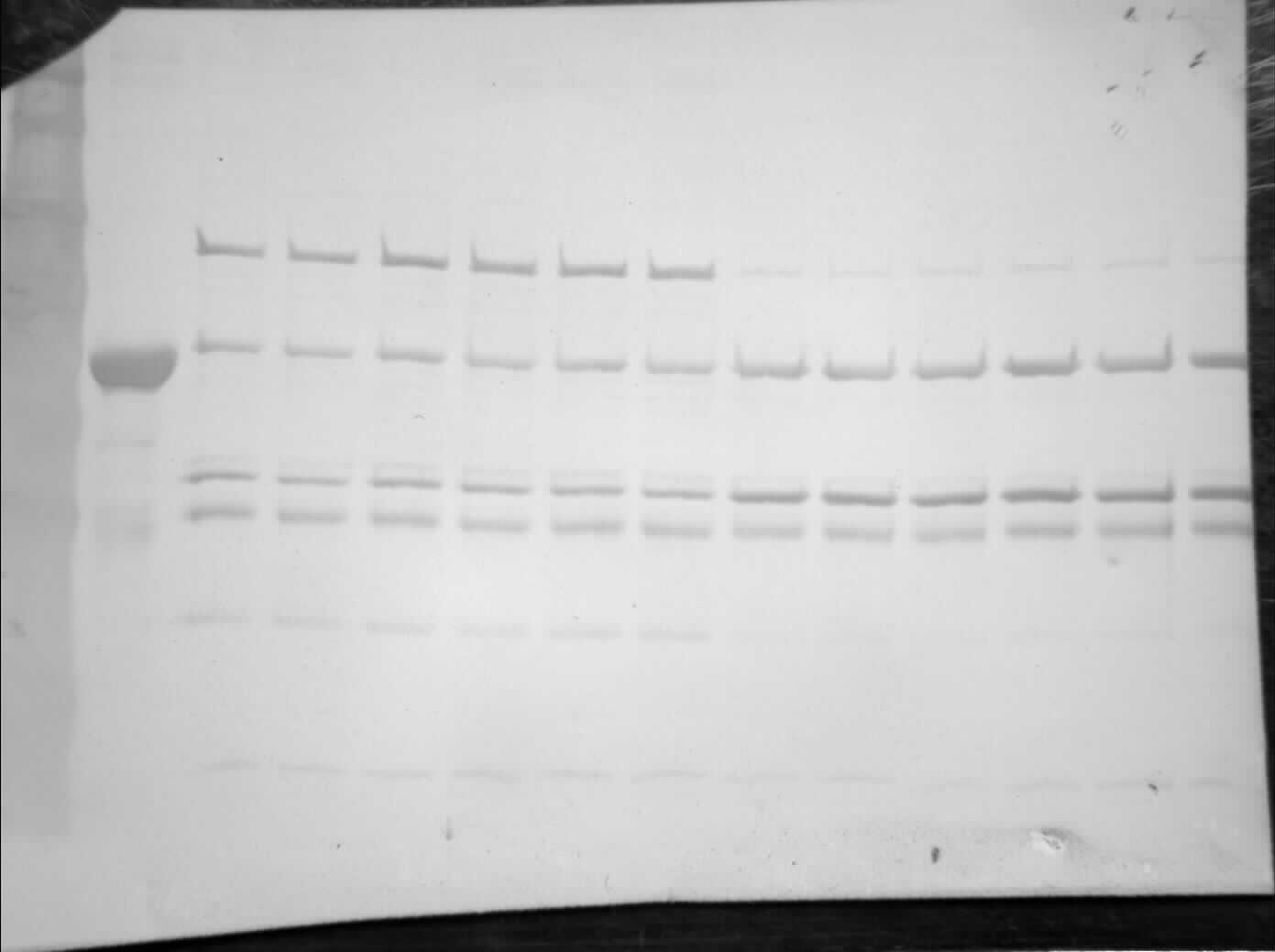

Supplement: Supplemental Information 13 [file peerj-06-4918-s013.jpg]

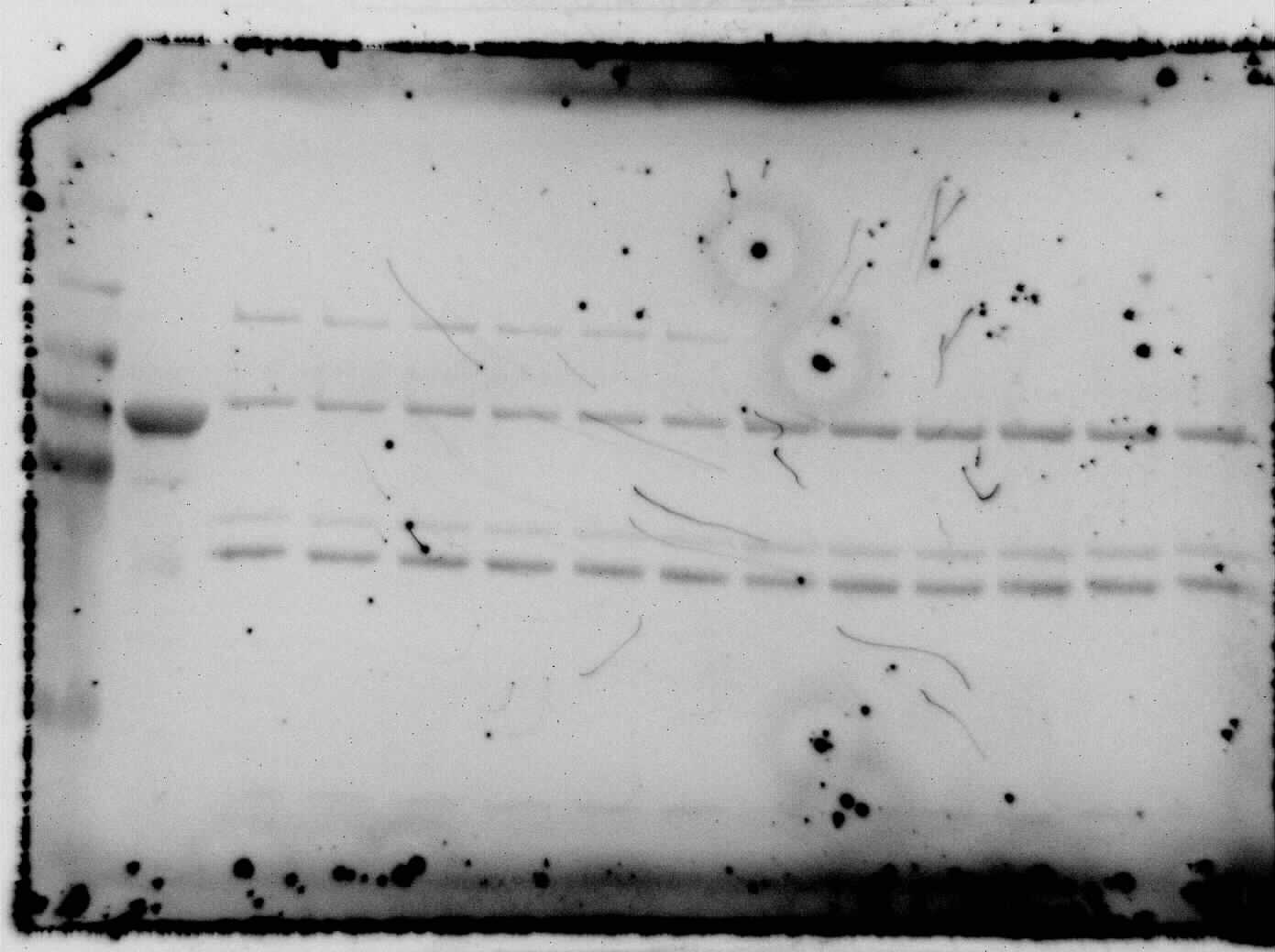

Supplement: Supplemental Information 14 [file peerj-06-4918-s014.jpg]

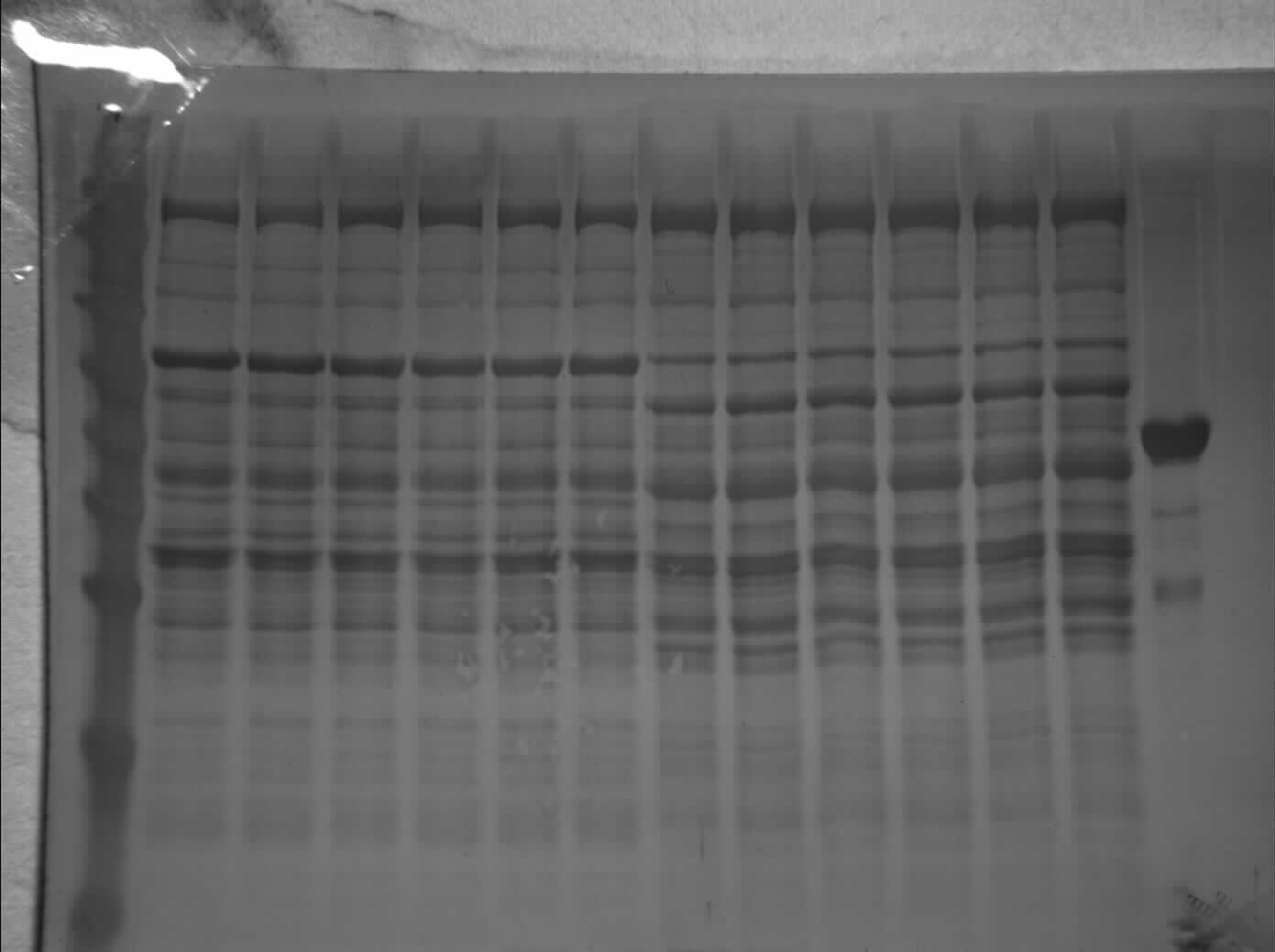

Supplement: Supplemental Information 15 [file peerj-06-4918-s015.jpg]

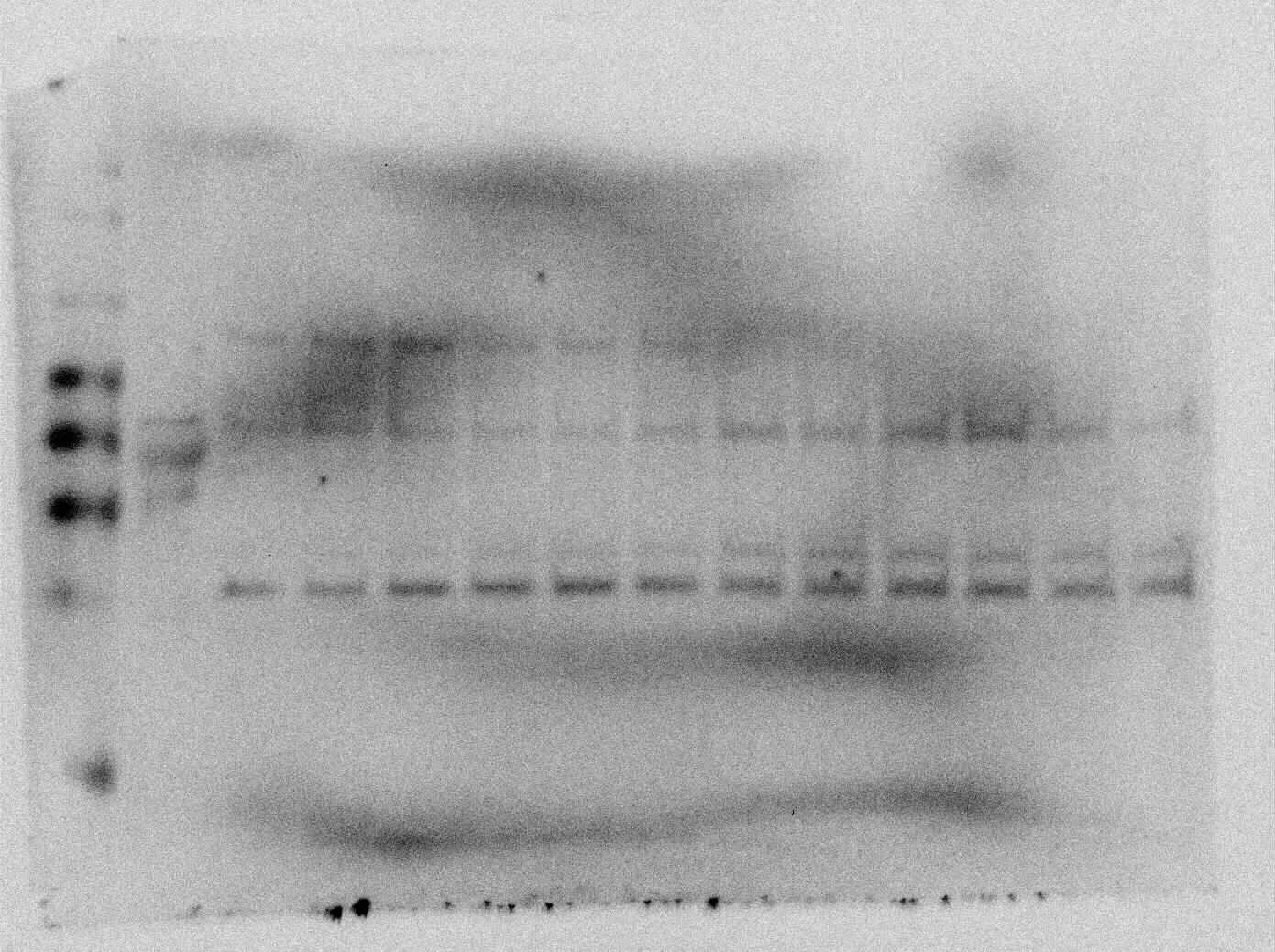

Supplement: Supplemental Information 16 [file peerj-06-4918-s016.jpg]

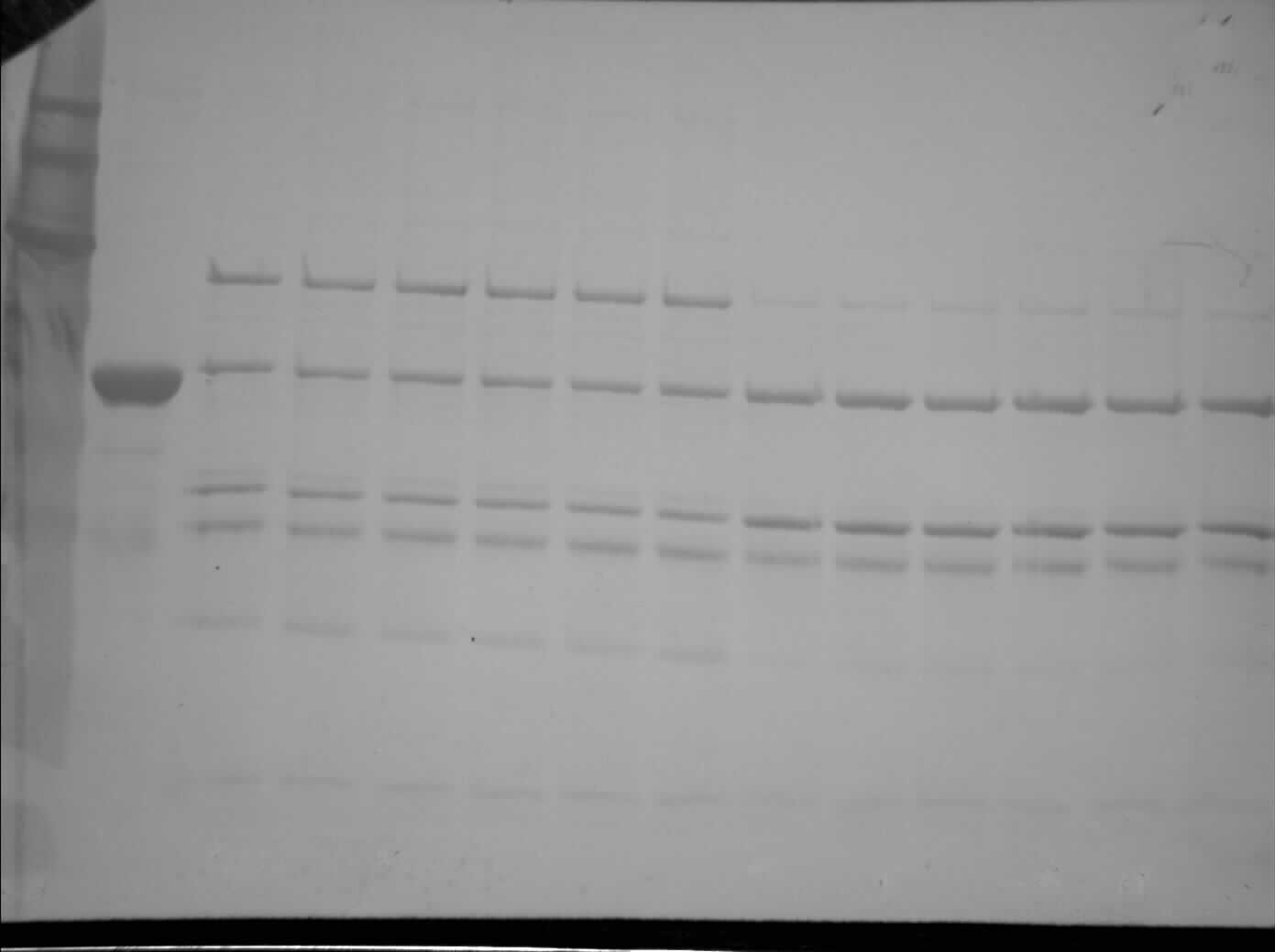

Supplement: Supplemental Information 17 [file peerj-06-4918-s017.jpg]
